# A versatile synthetic route for the preparation of titanium metal–organic frameworks†
†Electronic supplementary information (ESI) available: Full details of sample preparation, characterizations and photocatalysis experiments. See DOI: 10.1039/c5sc03620h


**DOI:** 10.1039/c5sc03620h

**Published:** 2015-11-02

**Authors:** Lanfang Zou, Dawei Feng, Tian-Fu Liu, Ying-Pin Chen, Shuai Yuan, Kecheng Wang, Xuan Wang, Stephen Fordham, Hong-Cai Zhou

**Affiliations:** a Department of Chemistry , Texas A&M University , College Station , Texas 77842-3012 , USA . Email: zhou@chem.tamu.edu; b Department of Material Science and Engineering , Texas A&M University , College Station , Texas 77843 , USA

## Abstract

Exploitation of new titanium metal–organic frameworks (Ti-MOFs) with high crystallinity has been attracting great attention due to their vast application potential in photocatalysis. Herein a versatile synthetic strategy, namely, High Valence Metathesis and Oxidation (HVMO), is developed to synthesize a series of Ti-MOFs with predesigned topologies and structures. The crystallinity of these Ti-MOFs was well maintained throughout, as confirmed by powder X-ray diffraction and gas adsorption measurements. Significantly, there were only a few examples of Ti-MOFs, not to mention a general synthetic strategy for various kinds of Ti-MOFs in the literature. This contribution also illustrates the intriguing potential of Ti-MOF platforms in photocatalysis.

## Abstract 2

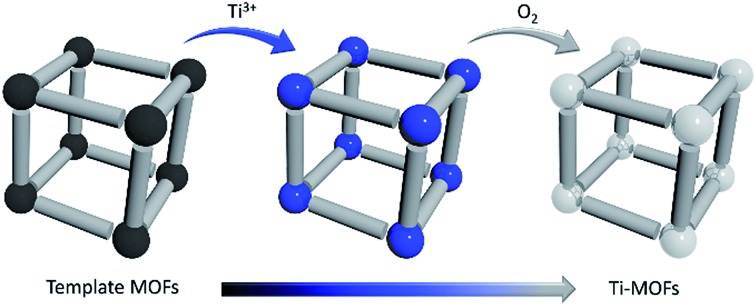
Through a versatile synthetic strategy, High Valence Metathesis and Oxidation (HVMO), a series of Ti-MOFs with predesigned topologies and structures were synthesized.

## Introduction

Over the past few years, metal–organic frameworks (MOFs) have attracted tremendous attention owing to their defined crystalline nature, pore tunability, structure diversity, as well as numerous potential applications such as gas adsorption,^1^ separation,^2^ catalysis,^3^ and sensing.^4^ Most of these applications require robust MOFs, making those constructed with high valent metals particularly desirable.^5^ While great efforts have been devoted to the development of MOFs containing trivalent metals, such as Fe^3+^, Cr^3+^, and Al^3+^,^6^ MOFs constructed with tetravalent metals are much less explored. Recently, the research on Zr-MOFs has flourished owing to the use of modulating reagents, which facilitate the growth of large crystals.^7^ However, titanium, even though in the same group as zirconium in the periodic table, has barely been adopted to construct MOFs despite its great abundance in the Earth's crust, low toxicity, and redox activity. Moreover, unlike zirconium clusters, which merely act as inorganic nodes to sustain the frameworks, titanium-oxo clusters in the previously reported Ti-MOFs can be viewed as TiO_2_ nanoparticles, endowing Ti-MOFs with additional photocatalytic properties. The integration of tunable functions on organic linkers with the photoactive inorganic nodes will turn Ti-MOFs into promising photocatalytic platforms.

Férey, Serre, and coworkers have initially demonstrated the preparation of titanium MOFs, MIL-91 8 and MIL-125.^9^ In particular, MIL-125 and its NH_2_-functionalized counterpart, MIL-125-NH_2_,^10^ showed great potential in light-driven hydrogen production^11^ and CO_2_ reduction applications.^12^ Recently, several other Ti-MOFs were reported, including NTU-9,^13^ PCN-22,^14^ Ti-MIL-101,^15^ and COK-69.^16^ However, Ti-MOFs have rarely been reported despite the burgeoning of many other high-valent-metal MOFs. Even for the few aforementioned Ti-MOFs, their synthetic conditions significantly diverged. Presumably, the following three major reasons may account for such synthetic difficulties for Ti-MOFs: (a) the high charge to radius (*Z*/*r*) value on Ti^4+^ results in strong coordination bonds between titanium nodes and the ligands. Accordingly, the poor reversibility of the metal–ligand bond association/dissociation process prevents the formation of crystalline products.^17^ (b) Most of the reactive titanium sources suffer from severe hydrolysis, which greatly limits the formation of Ti-MOFs. (c) Most of the known titanium carboxylates exhibit low symmetry or unfavorable connectivity, hindering the easy formation of periodic networks with the organic linkers.^18^

**Fig. 1:**
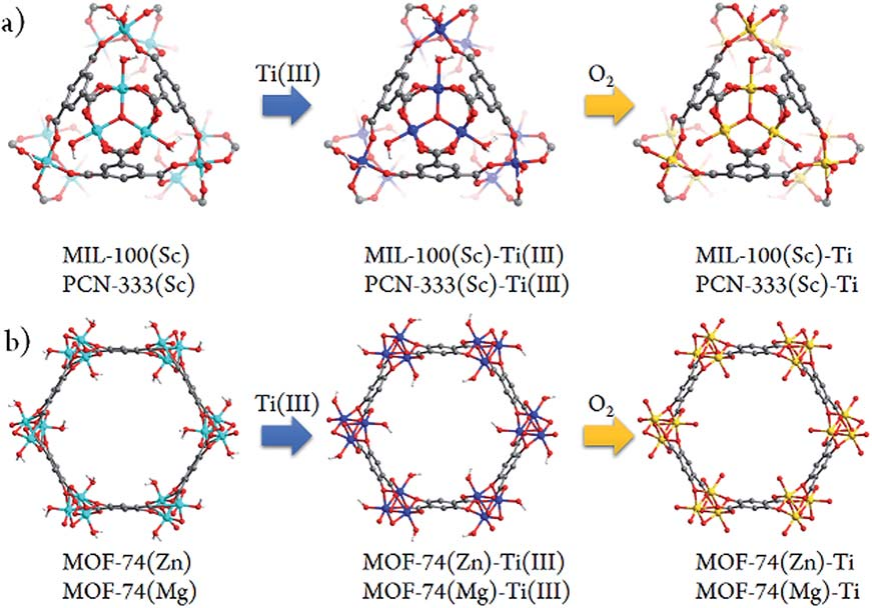
Schematic illustration of the stepwise HVMO procedure for the design of Ti-MOFs from the template MOFs: (a) MIL-100(Sc) and PCN-333(Sc) metal metathesis with Ti(iii), followed by metal node oxidation in the air (PCN-333(Sc) and MIL-100(Sc) have same topology but different structures); (b) similar process for MOF-74(Zn) and MOF-74(Mg).

Besides significant difficulties in direct synthesis, the Ti-MOFs synthesized directly usually don't have the predicted structures and topologies. Moreover, as the photocatalytic properties of Ti-MOFs can be greatly affected by the titanium oxo building units, the exploration of new synthetic approaches to obtain various Ti-MOF materials is critical. Herein, we present a general synthetic strategy, High Valence Metathesis and Oxidation (HVMO), to obtain Ti(iv) MOFs: starting from judiciously selected template frameworks, PCN-333(Sc),^17^ MIL-100(Sc),^19^ MOF-74(Zn),^20^ and MOF-74(Mg),^21^ we successfully synthesized a series of porous photoactive titanium MOFs, PCN-333(Sc)-Ti, MIL-100(Sc)–Ti, MOF-74(Zn)–Ti and MOF-74(Mg)–Ti, which is accomplished through Sc(iii) to Ti(iii) or Zn(ii)/Mg(ii) to Ti(iii) metathesis first, followed by a mild and effective oxidation step to Ti(iv) (Fig. 1). Generally, high valence metathesis is difficult and rare, thus its application in MOF preparation represents a significant step forward in Ti-MOFs synthesis. The Ti-MOFs made *via* HVMO not only maintain their crystallinity throughout the whole synthetic process, but also demonstrate excellent photocatalytic property.

## Results and discussion

Postsynthetic metathesis emerged as an alternative synthetic strategy due to its exceptional capability of obtaining certain MOFs that cannot or hardly be achieved directly.^22^ S. Sevov and coworkers have initially demonstrated the successful use of oxidizing Ti(iii) centers to obtain Ti(iv) open frameworks.^23^ Recently, the feasibility of postsynthetic exchange of Ti(iv) into MOFs has been demonstrated by Cohen^24^ and Dincă's groups.^25^ Moreover, HVMO strategy is built upon our previously reported post-synthetic metathesis and oxidation (PSMO) strategy.^26^ In the metathesis step of our strategy, starting from the divalent metal species, it is surprising to see that the trivalent metal species were successfully exchanged into the template MOFs, which is not possible as demonstrated by PSMO method. More importantly, we would be able to get the trivalent metal species intermediate MOFs even starting from the supposedly stable trivalent metal based template MOFs. The prerequisites to successfully utilize the HVMO strategy are carefully examined, both for the targeting metal species and for the template MOFs.

The prerequisites for the targeting metal species (Ti(iv) as an example) in the HVMO approach include: first, the targeting high valent metal species have to exhibit a suitable reduced state that can be conveniently achieved. It should be pointed out that during the synthesis of the Ti-MOFs, a pure titanium(iii) source with good stability under exchange environment is crucial. In our case, the commercially available, titanium(iii) chloride tetrahydrofuran complex (1 : 3), TiCl_3_(THF)_3_, was utilized as the titanium(iii) source. Second, the reduced species in lower oxidation state can undergo metal metathesis with a considerable rate to guarantee the framework crystallinity. The reduced state Ti(iii), with lower charge and larger radius, undergoes a ligand dissociation rate around 10^5^ sec^–1^ in the aqueous media, which is even faster than some M(ii) transition metal species in the third period, such as Ni(ii).^27^ Third, the reduced species can survive under the exchanging environment. Ti(iii)/Ti(iv) has higher redox potential than Cr(ii)/Cr(iii) in similar coordination environment of weak field ligands, making Ti(iii) easier to handle than Cr(ii), which is used in PSMO method, during the exchange process. Ti(iv) meets all these prerequisites, which enables HVMO strategy promising to synthesize Ti-MOFs.

Meanwhile, the template MOFs were also selected with careful consideration. Firstly, the metal species in the template MOFs cannot be reduced by Ti(iii). Otherwise, Ti(iii) would be oxidized to strong Lewis acidic Ti(iv) while the metals in the template MOFs would be reduced to lower valent metal ions. The lower valent metal based MOFs would be easily damaged by strong Lewis acidic Ti(iv) species. To verify this necessity, we conducted metathesis with PCN-333(Fe), which totally dissolved in one minute after we added Ti(iii) species (ESI, Section 4†). Secondly, all the template MOFs should contain open metal sites, which are occupied by the weakly coordinated neutral solvent molecules. These open metal sites could effectively accelerate the metathesis rate. Thirdly, the metal species in the template should possess a similar coordination environment with the targeting metal species. In this case, they are all six-coordinated. Last but not least, metal–ligand bonds in template MOFs should have certain lability to drive the metal exchange to completion. Otherwise, the exchange time would be very long and the crystallinity will be decreased during this process. With all these considerations in mind, we have chosen PCN-333(Sc), MIL-100(Sc), MOF-74(Zn), and MOF-74(Mg) as our template MOFs.

It is believed that HVMO strategy demonstrated in this study greatly facilitates the development of the original PSMO method as a general yet powerful strategy for synthesizing novel MOFs which cannot be obtained using the conventional method. And as expected, a series of highly porous Ti-MOFs were obtained using this HVMO strategy, while the direct synthesis using Ti(iii) and the corresponding linkers under solvothermal and anaerobic conditions only resulted in amorphous powders. The typical synthetic process of PCN-333(Sc)–Ti is: first, after washing with anhydrous *N*,*N*-dimethylformamide (DMF), the as-synthesized crystals were transferred into the glove box, where TiCl_3_(THF)_3_ was added, resulting an evident color change of the crystals from white to purple in three minutes. In order to facilitate this metathesis process, the crystals were sealed in a vial and kept at 120 °C for 24 hours. Meantime, the mother liquid was refreshed every 6 hours. After reaching the exchange equilibrium, the excess TiCl_3_ molecules were removed by washing thoroughly with DMF, yielding dark purple crystals, PCN-333(Sc)–precursor. Then the sample was treated with oxygen/water-free methanol for three days and activated at 150 °C for 5 hours. The activated sample was oxidized in the air causing an apparent color change from dark purple to white as shown in Fig. S2.† Using this dry oxidation method, we would be able to keep the MOFs' crystallinity to the highest level. The synthetic procedure was similar for the other three Ti-MOFs and the obvious color changes were also observed, which clearly indicated the metathesis step and the oxidation step (ESI, Section 5†). Moreover, the microscopy photos before and after metal exchange demonstrated that both the crystal shape and size did not change, indicating that HVMO process is a true crystal-to-crystal process not involving any dissolution or recrystallization. In order to confirm the oxidation state of final Ti-MOFs, we performed X-ray photoelectron spectroscopy (XPS) measurements for all the Ti-MOFs (ESI, Section 12†). The Ti2p_3/2_ signal at 458.7 eV and Ti2p_1/2_ signal at 464.2 eV confirmed that the oxidation state of Ti ions in the frameworks is +4, which is also consistent with the color changes observed during the exchange process.

The atomic ratio of titanium in these Ti-MOFs was analyzed using inductively coupled plasma mass spectrometry (ICP-MS) and energy-dispersive X-ray spectroscopy (EDS) analyses (Table 1). The metal exchange ratio of PCN-333(Sc)–Ti is 85.9%, which is much higher than the 52.0% of MIL-100(Sc)–Ti. Two possible reasons may account for this. Firstly, although PCN-333(Sc) possesses the same inorganic building block with MIL-100(Sc), the longer linker in PCN-333(Sc) endows the lattice with higher flexibility to facilitate the metal exchange completeness.^28^ Secondly, the larger ligand gives rise to larger pores and windows in PCN-333(Sc), which further enhance the diffusion rate inside the MOF materials. An exchange ratio of 100% was finally achieved for the MOF-74(Zn)–Ti while starting with MOF-74(Mg), a much lower exchange ratio, 35.1%, was observed. We attempted to attribute the difference to two reasons: first, the Mg(ii) framework is inherently more stable arising from the stronger metal to ligand bond compared to the Zn(ii) isostructure, which is less likely to undergo efficient metathesis. Second, while Zn(ii) has the almost same radius with Ti(iii), there is a big difference between Mg(ii) and Ti(iii). This great difference will increase the frameworks tension during the exchange, which hinders the exchange rate. To the best of our knowledge, MOF-74(Zn)–Ti is a rare example of a MOF with a one dimensional metal chain that undergoes the complete metal exchange.

**Table 1:** EDS and ICP-MS analysis for titanium MOFs

Ti%^*a*^	333(Sc)–Ti	100(Sc)–Ti	74(Zn)–Ti	74(Mg)–Ti
EDS	85.9%	52.0%	100%	35.1%
ICP-MS	88.0%	48.8%	94.7%	37.9%

^*a*^Atomic percentage.

**Fig. 2:**
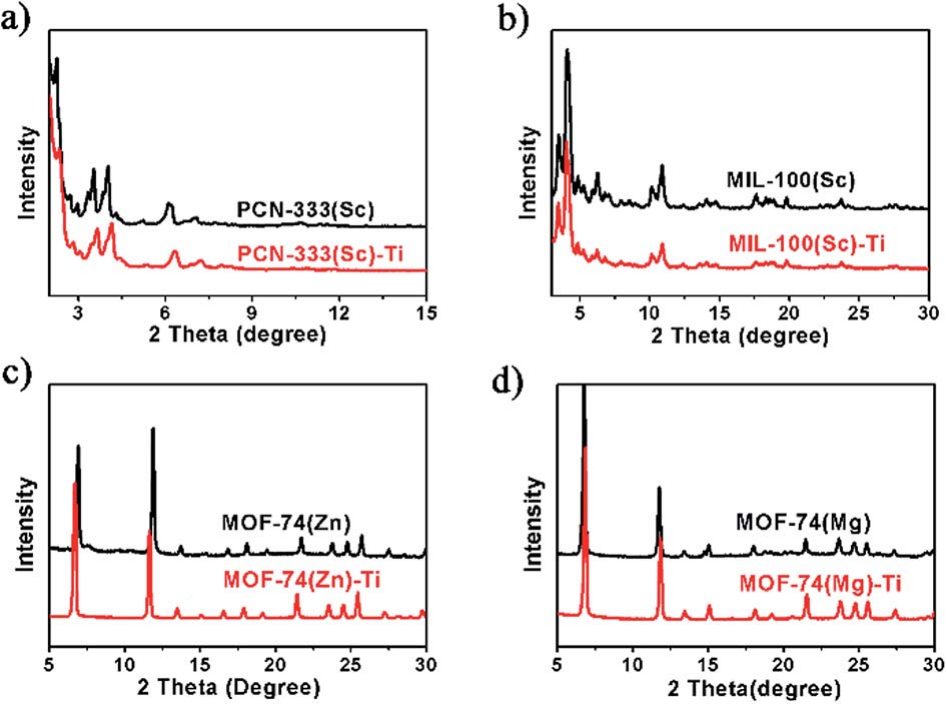
PXRD patterns for template MOFs and the corresponding titanium MOFs.

Power X-ray diffraction (PXRD) patterns of the MOFs before and after metal exchange well coincide with each other, indicating the obtained products have the same structure with templates and the crystallinity is well-maintained during the HVMO process (Fig. 2). In order to further confirm the crystal structure as well as the crystallinity, high resolution synchrotron PXRD data of MOF-74(Zn), MOF-74(Zn)–Ti, MOF-74(Mg) and MOF-74(Mg)–Ti was collected (Fig. S10 and S11†), and Rietveld refinement was performed to refine the crystal structure of each MOF. It confirmed that MOF-74(Zn)–Ti has almost the same unit cell with MOF-74(Zn), and MOF-74(Mg)–Ti has almost the same unit cell with MOF-74(Mg) (Table S1†), proving that MOF-74(Zn)–Ti and MOF-74(Mg)–Ti have the same crystal structure with the corresponding templating MOF-74.

**Fig. 3:**
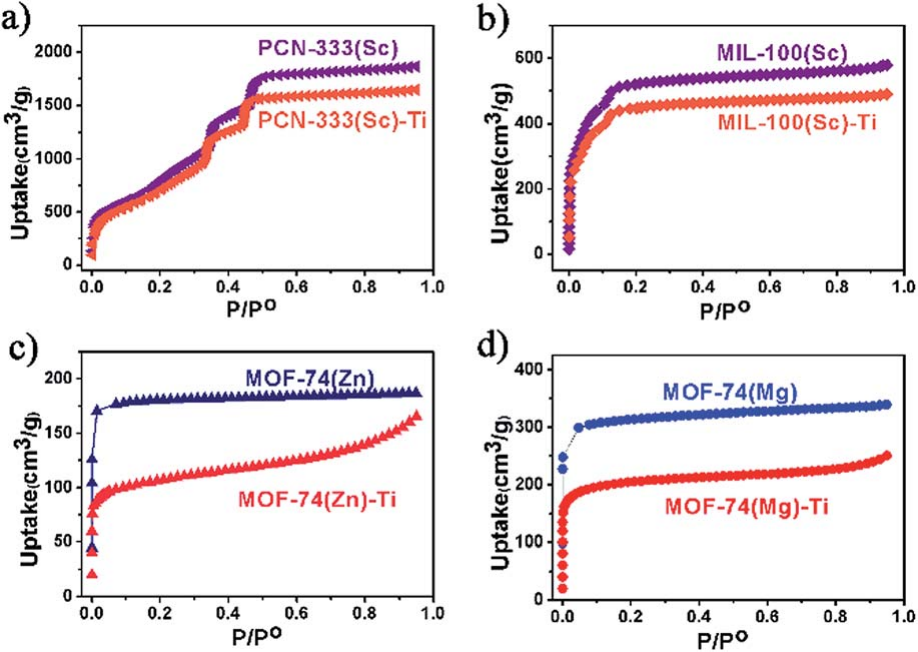
N_2_ uptakes for template MOFs and the corresponding titanium MOFs at 77 K, 1 atm.

N_2_ adsorption measurements were also conducted to evaluate the crystallinity of these Ti-MOFs (Fig. 3). As can been observed from the N_2_ adsorption isotherms, the porosity of the Ti-MOFs is kept almost intact compared to their corresponding template MOFs. The small decrement could be ascribed to the extra anions that are required for the charge balance since Ti(iv) has higher oxidation state, resulting in higher crystal density. Accordingly the gravimetric adsorption of Ti-MOFs should be smaller than the template MOFs.

We tested the water stability of our Ti-MOFs. From the PXRD results, we can tell that MIL-100(Sc)–Ti, MOF-74(Zn)–Ti, and MOF-74(Mg)–Ti can stay in pure water for 18 h without losing their crystallinity. PCN-333(Sc)–Ti only maintains its crystallinity in pure water for 3 h probably due to the much larger ligand and porosity. Such extended linker effects on the framework stability have also been reported in many other MOF systems, such as UiO-66, 67, 68.^29^

**Fig. 4:**
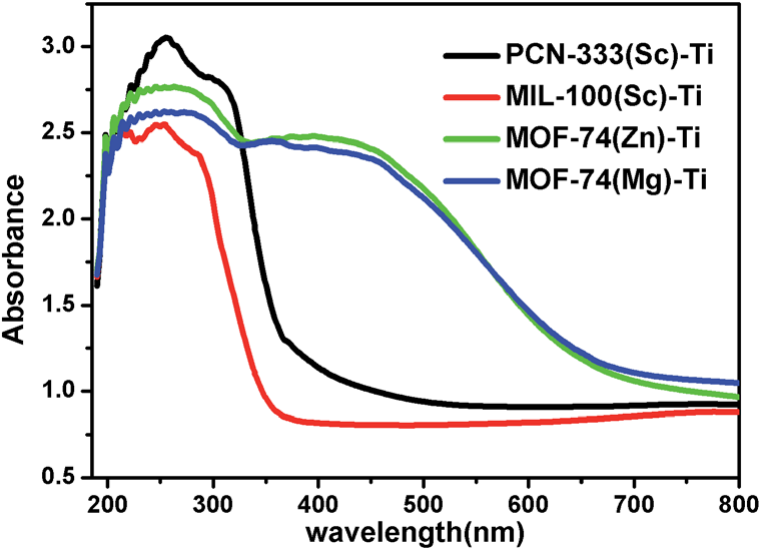
UV/Vis absorption spectra of PCN-333(Sc)–Ti (black), MIL-100(Sc)–Ti (red), MOF-74(Zn)–Ti (green) and MOF-74(Mg)–Ti (blue).

With diverse titanium-oxo clusters exchanged into the frameworks, we investigated the optical properties of these Ti-MOFs using diffuse reflectance UV-Vis absorption spectroscopy (Fig. 4). Compared with the absorption edge at 380 nm of PCN-333(Sc)–Ti and MIL-100(Sc)–Ti, MOF-74(Zn)–Ti and MOF-74(Mg)–Ti show an extra absorption band centered at about 450 nm with the absorption edge extended to around 660 nm. In particular, the photocurrent profile of MOF-74(Zn)–Ti indicates that it is active under visible light (>450 nm) illumination (Fig. S30†). In Mott–Schottky measurement, the positive slope of the obtained *C*^–2^ to potential is consistent with typical n-type semiconductor, as is our material (Fig. S31†). Meanwhile, there are obvious color differences between these titanium MOFs (Fig. S2–S5†): PCN-333(Sc)–Ti and MIL-100(Sc)–Ti are white, MOF-74(Mg)–Ti is orange, while MOF-74(Zn)–Ti is dark red. Such differences can be probably ascribed to two reasons. On one hand, PCN-333(Sc)–Ti and MIL-100(Sc)–Ti are composed of trinuclear clusters while MOF-74(Zn)–Ti and MOF-74(Mg)–Ti are composed of one dimensional titanium chains, which result in distinguished differences of HOMO–LUMO gaps. On the other hand, as a good electron donation moiety, 2,5-dioxido-1,4-benzenedicarboxylate (DOBDC) would provide effective ligand to metal charge transfer in Ti-MOF-74 structure, resulting in visible light absorption.

**Fig. 5:**
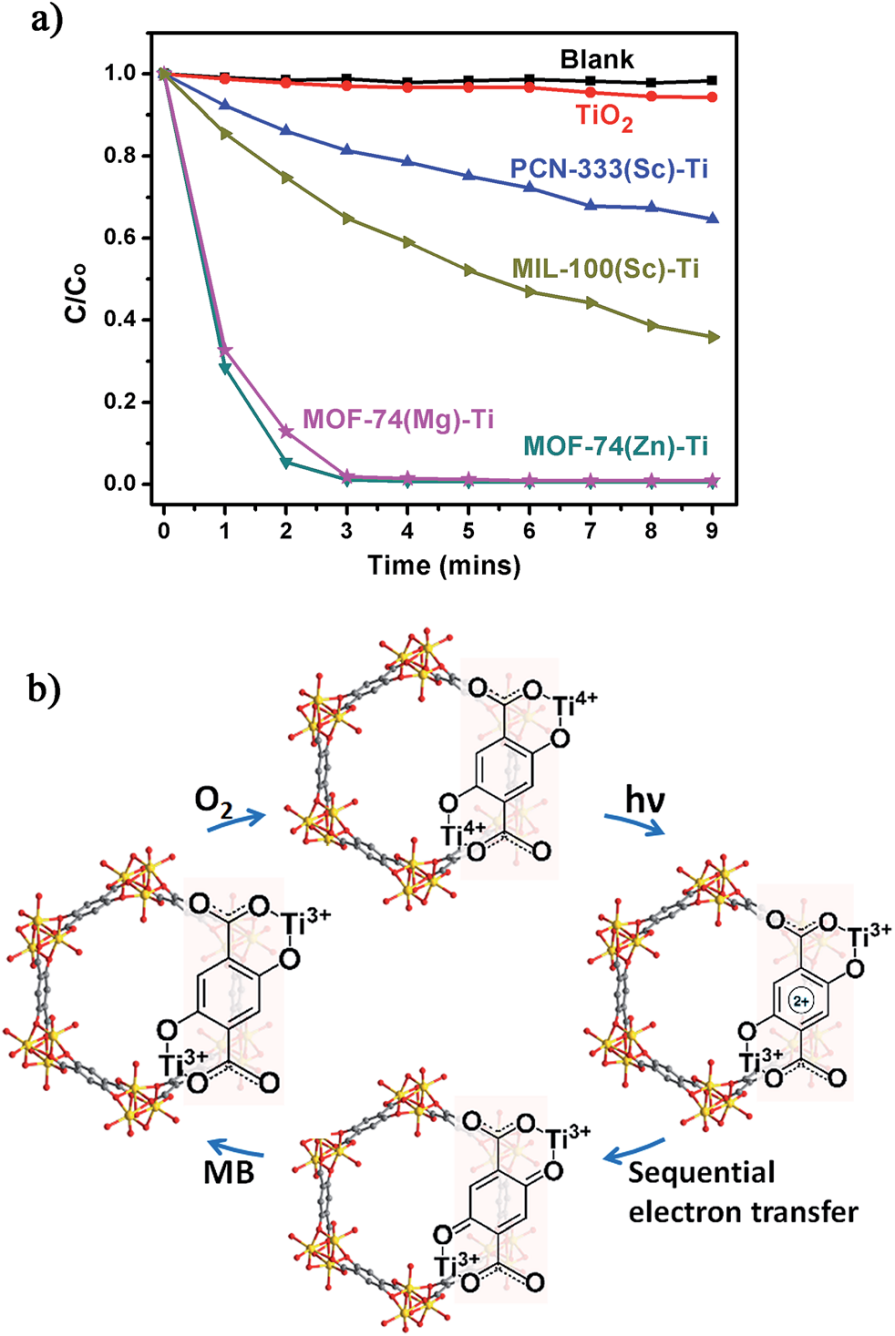
(a) Photodegradation of MB using no catalyst (blank), TiO_2_, PCN-333(Sc)–Ti, MIL-100(Sc)–Ti, MOF-74(Zn)–Ti and MOF-74(Mg)–Ti with 300 W xenon light irradiation; (b) proposed mechanism of MB degradation of Ti-MOF-74 in the presence of air.

The photocatalytic potential of the Ti-MOFs was examined using the photodegradation of methylene blue (MB) as a probe reaction. 15 mg of catalyst was suspended in 15 mL of 500 μM aqueous MB solution without addition of hydrogen peroxide. The solution was stirred in the dark for 2 hours to achieve the adsorption equilibrium before being illuminated with a 300 W Xe lamp for nine minutes. The concentration change of MB was monitored by measuring the optical absorption at 660 nm of the suspension at regular intervals (Fig. 5a). In our control experiment (without any catalyst, blank line), there was no degradation of MB in such a short time. The photo-degradation of MB in the presence of TiO_2_ was relatively slow, with less than 6% of MB was degraded and this ratio increased to 35% and 64% using PCN-333(Sc)–Ti and MIL-100(Sc)–Ti as catalysts. The better catalytic efficiency of MIL-100(Sc)–Ti compared with PCN-333(Sc)–Ti could be ascribed to the better water stability. Both MOF-74(Zn)–Ti and MOF-74(Mg)–Ti revealed excellent catalytic efficiencies, with conversions up to 98% after only three minutes. All the Ti-MOFs showed much better catalytic performance than TiO_2_ as well as the corresponding template MOFs (Fig. S32–S35†), indicating the Ti content in our Ti-MOFs indeed played a vital role in the photocatalytic process.

The excellent photocatalytic performance of Ti-MOF-74 can be attributed to its capability of absorbing a broader range of the irradiating light as proved by the diffuse reflectance UV-Vis absorption spectroscopy and a longer excitation lifetime. To illustrate photodegradation of MB within the system of Ti-MOF-74, we herein present a proposed mechanism (Fig. 5b): in this photochemical reaction, Ti-MOF-74 acts as the chromophore. Upon excitation, electrons transfer from DOBDC to the Ti-oxo clusters, reducing Ti^4+^ into Ti^3+^ ions, which separates the electron–hole couple. Meanwhile the oxidized DOBDC is stabilized by the formation of the thermodynamic product, a benzoquinone species, presumably by a sequential electron transfer step.^30^ The benzoquinone species can be reduced by highly reductive MB, which is followed by the oxidation of Ti^3+^ ions by O_2_, fulfilling the catalytic cycle. The formation of Ti^3+^ and benzoquinone intermediates efficiently prevents the electron–hole recombination, which inherently accelerates the photocatalytic activity of Ti-MOF-74. With organic ligands as dye, inorganic clusters as photoactive TiO_2_ nanoparticles, high porosity and large one dimensional channels, Ti-MOF-74 has great potential for practical applications in the photocatalysis.

## Conclusions

In summary, we have successfully demonstrated a versatile synthetic strategy, HVMO, towards various titanium MOFs, PCN-333(Sc)–Ti, MIL-100(Sc)–Ti, MOF-74(Zn)–Ti and MOF-74(Mg)–Ti, which exhibit high porosity and excellent photocatalytic activity. The crystallinity is well maintained during the HVMO process as confirmed by nitrogen uptake and PXRD measurements. This study manifests an effective method to explore promising Ti-MOF platforms for photocatalytic applications.

## Experimental

### The synthesis of template MOFs and the corresponding titanium MOFs

#### Synthesis of PCN-333(Sc)

4,4′,4′′-*s*-Triazine-2,4,6-triyl-tribenzoic acid (TATB, 80 mg) and ScCl_3_·6H_2_O (200 mg) were dissolved in 10 mL DMF in a 20 mL vial. The mixture was heated up in 150 °C oven for 2 hours until white precipitate formed. The white precipitate was centrifuged and washed with fresh DMF several times. Yield (based on ligand): ∼90%.

#### Synthesis of PCN-333(Sc)–Ti

30 mg as-synthesized PCN-333(Sc) was washed with dry DMF several times. The mixture was bubbled with nitrogen for 15 min, and then transferred into the glove box when 50 mg TiCl_3_(THF)_3_ was added. The crystals' color obviously changed from white to purple in 3 min. In order to make exchange complete, the reaction was allowed to continue at 120 °C for 24 hours. Meantime, the mother liquid was exchanged with fresh TiCl_3_(THF)_3_ DMF solution every 6 hours. The solid was washed with oxygen/water-free DMF several times to afford PCN-333(Sc)–precursor. The precursor was solvent exchanged with oxygen/water free methanol for 3 days before being activated at 150 °C for 5 hours. After this, the material was exposed to air to get oxidized to PCN-333(Sc)–Ti. The color changed from purple to white.

#### Synthesis of MIL-100(Sc)

1,3,5-Benzenetricarboxylate (BTC, 60 mg) and ScCl_3_·6H_2_O (200 mg) were dissolved in 10 mL DMF. The mixture was heated up in 150 °C oven for 2 h until white precipitate formed. The white precipitate was centrifuged and washed with fresh DMF several times. Yield (based on ligand): ∼85%.

#### Synthesis of MIL-100(Sc)–Ti

As-synthesized 30 mg MIL-100(Sc) was washed with dry DMF three times. The mixture was bubbled with nitrogen for 15 min, and then transferred into glove box where 50 mg TiCl_3_(THF)_3_ was added. The crystals' color obviously changed to brown in 10 min. In order to facilitate the exchange rate, the reaction was allowed to continue at 120 °C for 24 hours. Meantime, the mother liquid was exchanged with fresh TiCl_3_(THF)_3_ DMF solution every 6 hours. The solid was washed with oxygen/water-free DMF to get MIL-100(Sc)–precursor. The precursor was solvent exchanged with oxygen/water-free methanol for 3 days before being activated at 150 °C for 5 hours. After this, the material was exposed to air to get oxidized to MIL-100(Sc)–Ti. The color changed from brown to white.

#### Synthesis of MOF-74(Zn)

Zn(NO_3_)_2_·6H_2_O (180 mg) and DOBDC (70 mg) were dissolved with 15 mL DMF in a 20 mL vial. The vial was sealed and sonicated for 5 minutes until the solid was completely dissolved. To this solution, 1 mL of ethanol followed by 1 mL of deionized water was added dropwise. The vial was sonicated resulting in a clear, yellow solution, which was heated in an isothermal oven at 100 °C for 24 h yielding yellow needle crystals MOF-74(Zn). Yield (based on ligand): ∼80%.

#### Synthesis of MOF-74(Zn)–Ti

As-synthesized 30 mg MOF-74(Zn) was washed with dry DMF several times and immersed in dry methanol for 3 days before being activated at 130 °C for 9 h to remove the terminal solvent molecules on the open metal sites. After activation, MOF-74(Zn) was transferred into the glove box when 50 mg TiCl_3_(THF)_3_ and 2 mL anhydrous DMF were added. In order to make the exchange complete, the reaction was allowed to continue at 100 °C for 18 hours. Meantime, the mother liquid was exchanged with fresh TiCl_3_(THF)_3_ DMF solution every 6 hours. The crystals' color changed from light yellow to dark purple. The solid was washed with fresh oxygen/water-free DMF to get MOF-74(Zn)–precursor. The precursor was solvent exchanged with oxygen/water-free methanol for 3 days before activated at 60 °C for 5 hours. After this, the material was exposed to air to get oxidized to dark red MOF-74(Zn)–Ti.

#### Synthesis of MOF-74(Mg)

Mg(NO_3_)_2_·6H_2_O (150 mg) and DOBDC (60 mg) were dissolved with 15 mL DMF in a 20 mL vial. The vial was sealed and sonicated for 5 minutes until the solid was completely dissolved. To this solution, 1 mL of ethanol and 1 mL of deionized water was added. The vial was sonicated resulting in a clear, light yellow solution. This solution was heated in an isothermal oven at 120 °C for 24 h yielding yellow needle crystals MOF-74(Mg). Yield (based on ligand): ∼75%.

#### Synthesis of MOF-74(Mg)–Ti

As-synthesized 30 mg MOF-74(Mg) was washed with dry DMF several times and immersed in dry methanol for 3 days before being activated at 130 °C for 9 h to remove the terminal solvent molecules on the open metal sites. The activated MOF-74(Mg) was transferred into the glove box when 60 mg TiCl_3_(THF)_3_ in 2 mL anhydrous DMF was added. In order to make the exchange complete, the reaction was allowed to continue at 120 °C for 36 hours. Meantime, the mother liquid was exchanged with fresh TiCl_3_(THF)_3_ DMF solution every 6 hours. The crystals' color changed from yellow to black. The solid was washed with fresh oxygen/water-free DMF to get MOF-74(Mg)–precursor. The precursor was solvent exchanged with oxygen/water-free methanol for 3 days before being activated at 60 °C for 5 hours. After this, the material was exposed to air to get oxidized to orange MOF-74(Mg)–Ti.

### Photodegradation of methylene blue with Ti-MOFs

The evaluation of photocatalytic activities of the samples for the photocatalytic degradation of organic dyes was performed at ambient temperature (25 °C). The procedure was as follows: 15 mg of sample was dispersed into 15 mL of methylene blue (MB) aqueous solution (500 μmol L^–1^). The mixture was stirred continuously with a magnetic stirring bar for two hours in the dark to reach the adsorption equilibrium. The photocatalytic dye degradation was carried out by irradiating the suspension with a 300 W xenon lamp. At different time intervals, analytical samples were withdrawn and analyzed by UV-Vis spectroscopy. The degradation efficiency was determined by dividing *C*/*C*_0_ with time, where *C* is the remained MB concentration and *C*_0_ is the starting MB concentration.

## Supplementary Material

Supplementary information
