# Supplementary material for: A versatile synthetic route for the preparation of titanium metal–organic frameworks
Source: Chem Sci. 2015 Nov 2;7(2):1063–9. doi: 10.1039/c5sc03620h (PMC5954845; doi:10.1039/c5sc03620h)
Supplement: Supplementary file 1 [file SC-007-C5SC03620H-s001.pdf]

Electronic Supplementary Material (ESI) for Chemical Science.
This journal is © The Royal Society of Chemistry 2015

Supporting Information

A Versatile Synthetic Route for the Preparation of Titanium Metal-Organic Frameworks

Lanfang Zou,[a] Dawei Feng,[a] Tian-Fu Liu,[a] Ying-Pin Chen,[b] Shuai Yuan,[a] Kecheng Wang,[a] Xuan Wang,[a] Stephen Fordham,[a] and Hong-Cai Zhou*[ab]

[a]Department of Chemistry, Texas A&M University, College Station, Texas 77843, United States

[b]Department of Materials Science and Engineering, Texas A&M University, College Station, Texas 77843, United States

*To whom correspondence should be addressed. Email: zhou@chem.tamu.edu

Tel: +1 (979) 845-4034; Fax: +1 (979) 845-1595

Contents

Section 1. Materials and Instrumentation 3

Section 2. The Synthesis Procedure for the Ligands. 4

Section 3: The Synthesis of Template MOFs and the Corresponding Titanium MOFs. 5

Section 4: Metal Exchange with PCN-333(Fe) as the Template 8

Section 5: Color Changes during HVMO Process. 9

Section 6: Powder X-ray Diffraction (PXRD) Patterns for Templates and Ti-MOFs. 11

Section 7: Powder Refinements for MOF-74(Zn)-Ti and MOF-74(Mg)-Ti. 12

Section 8: Porosity Measurements for the Template MOFs and Ti-MOFs. 15

Section 9. Energy Dispersive X-ray Spectroscopy (EDS) and Inductively Coupled Plasma Emission-Mass Spectrometry (ICP-MS) Analysis. 17

Section 10. Water Stability Test for Ti-MOFs. 19

Section 11. Optimization of Exchange Conditions for PCN-333(Sc)-Ti. 21

Section 12. X-ray Photoelectron Spectroscopy (XPS) Analysis of Ti-MOFs. 23

Section 13. Photoabsorption Performance of Ti-MOFs. 25

Section 14. The Photoelectrochemical Properties of MOF-74(Zn)-Ti. 26

Section 15. Photodegradation of Methylene Blue with Ti-MOFs. 28

Section 16. PXRD Patterns of Ti-MOFs after MB Photodegradation. 28

Section 17. Thermogravimetric Analysis. 31

Section 1. Materials and Instrumentation

Materials

N, N-dimethylformamide (DMF), acetone, methanol, toluene, chloroform ($CHCl_3$), 2,4,6-tricholoro-1,3,5-trizaine, anhydrous aluminum chloride ($AlCl_3$), acetic acid, chromium oxide, scandium(III) chloride hexahydrate ($ScCl_3$•$6H_2O$), magnesium nitrate hexahydrate ($Mg(NO_3)_2$•$6H_2O$), zinc nitrate hexahydrate ($Zn(NO_3)_2$•$6H_2O$), 1,3,5-benzenetricarboxylate (BTC), 2,5-dihydroxyterephthalic acid (DOBDC), titanium(III) chloride tetrahydrofuran complex (1:3) $TiCl_3(THF)_{3,}$ TiO_2 were purchased from Alfa Aesar. All commercial chemicals were used without further purification unless otherwise mentioned.

Instrumentation

Powder X-ray diffraction (PXRD) was carried out with a BRUKER D8-Focus Bragg-Brentano X-ray Powder Diffractometer equipped with a Cu sealed tube (λ = 1.54178) at 40 kV and 40 mA. Thermogravimetric analyses (TGA) were carried out on a Shimadzu TGA-50 thermal analyzer from room temperature to 700 °C at a ramp rate of 3 °C/min in a flowing nitrogen atmosphere. Nuclear magnetic resonance (NMR) data were collected on a Mercury 300 spectrometer. Gas sorption measurements were conducted using a Micrometritics ASAP 2020 system at different temperatures. Energy dispersive X-ray spectroscopy (EDS) was carried out by JEOL JSM-7500F with Oxford EDS system equipped with X-ray mapping. Inductively Coupled Plasma Emission - Mass Spectrometry (Laser Ablation) (ICP-MS) was carried out by Perkin Elmer DRCII ICP-MS with both solution and laser ablation capabilities. UV-Vis diffuse reflectance spectra (UV-Vis DRS) were obtained using a UV-Vis-NIR spectrophotometer (Hitachi U-4100) in which barium sulfate

($BaSO_4$) powder was used as a reference. The UV-Vis absorption spectra were recorded on a Shimadzu UV-2450 spectrophotometer. The high resolution synchrotron PXRD data was collected at beamline 17-BM at the Advanced Photon Source, Argonne National Laboratory using the wavelength of 0.72768 Angstrom. X-ray photoelectron spectroscopy was carried out by Kratos Axis Ultra Imaging X-ray photoelectron spectrometer using Mg X-ray source.

Section 2. The Synthesis Procedure for the Ligands.

Synthesis of 4, 4′, 4″-s-triazine-2,4,6-triyl-tribenzoic acid (H_3TATB)

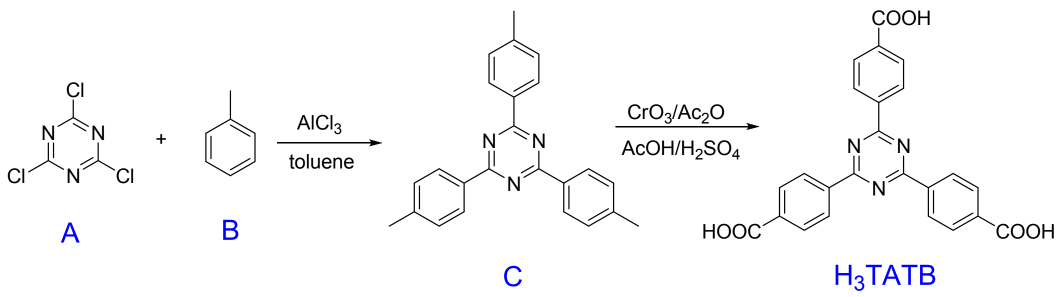

Figure S1. Synthetic procedure of **H_3TATB**

(1) *Synthesis of* **C**

20g $AlCl_3$ is added into a 250 ml three necked flask containing 50 ml dry toluene. The temperature is increased to 60 °C. 8.3g $C_3N_3Cl_3$ is then added in portions of about 0.2g at a time for about 1 hour. After all the $C_3N_3Cl_3$ is added, the mixture is allowed to stir for one more hour (or overnight). Then the resulted red sticky oil is poured into a large amount of ice water to kill the catalyst. 100 ml $CHCl_3$ is added. The water layer is decanted, and the organic layer is filtered. Methanol is added into $CHCl_3$ to precipitate some needle-like solid. The rest of the solid is

recrystallized with hot toluene to afford white, needle-like crystalline solids (dissolve solid in hot toluene, then place in refrigerator). ^{1}H NMR (300 MHz, $CDCl_3$): δ = 2.46 (s, 9 H), 7.35 (d, 6 H), 8.64 (d, 6 H) ppm.

(2) *Synthesis of* **H_3TATB**

A 500 mL three-necked flask was charged with 2.78 g **C** dissolved in 70 mL acetic acid, then 4.4 mL of H_2SO_4 was added. 7.2 g chromium oxide was dissolved in 4.8 mL acetic anhydride with stirring, then carefully added into the solution slowly, using a cold water-ice bath to keeping the temperature below 50 °C. The resulting black-brown slurry was stirred overnight. Pour the reaction mixture into 300 mL cold water, stir 1 hour to well mixed, and then filter. The solid was washed with water to remove the chromium acid. Dissolve the white solid in 200 mL 2N NaOH solution. After the unreacted starting material was removed by filtration, the solution was acidified with 10% HCl solution to give a white, crude product precipitate (until pH<3). Filter and dry the crude product. Recrystallization from DMF gave pure product as a white solid. ^{1}H NMR (300 MHz, DMSO): δ = 8.20 (d, 6 H), 8.85 (d, 6 H), 13.35 (s, 3 H) ppm.

Section 3: The Synthesis of Template MOFs and the Corresponding Titanium MOFs.

Synthesis of **PCN-333(Sc)**[1]: TATB (80 mg) and $ScCl_3.6H_2O$ (200 mg) were dissolved in 10mL DMF in a 20 mL vial. The mixture was heated up in 150 °C oven for 2 hours until a white precipitate

formed. The white precipitate was centrifuged and washed with fresh DMF several times. Yield (based on ligand): ~90%.

Synthesis of **PCN-333(Sc)-Ti:** 30 mg as-synthesized **PCN-333(Sc)** was washed with dry DMF several times. The mixture was bubbled with nitrogen for 15 min, and then transferred into the glove box when 50 mg $TiCl_3(THF)_3$ was added. The crystals' color obviously changed from white to purple in 3 min. In order to make exchange complete, the reaction was allowed to continue at 120 ^oC for 24 hours. Meantime, the mother liquid was exchanged with fresh $TiCl_3(THF)_3$ DMF solution every 6 hours. The solid was washed with oxygen/water-free DMF several times to afford **PCN-333(Sc)-precursor**. The precursor was solvent exchanged with oxygen/water free methanol for 3 days before being activated at 150 ^oC for 5 hours. After this, the material was exposed to air to get oxidized to **PCN-333(Sc)-Ti.** The color changed from purple to white.

Synthesis of **MIL-100(Sc)**[2]: BTC (60 mg) and $ScCl_3.6H_2O$ (200 mg) were dissolved in 10 mL DMF. The mixture was heated up in 150^oC oven for 2 h until white precipitate formed. The white precipitate was centrifuged and washed with fresh DMF several times. Yield (based on ligand): ~85%.

Synthesis of **MIL-100(Sc)-Ti:** As-synthesized 30 mg **MIL-100(Sc)** was washed with dry DMF three times. The mixture was bubbled with nitrogen for 15 min, and then transferred into glove box where 50 mg $TiCl_3(THF)_3$ was added. The crystals' color obviously changed to brown in 10 min. In order to facilitate the exchange rate, the reaction was allowed to continue at 120 ^oC for 24 hours. Meantime, the mother liquid was exchanged with fresh $TiCl_3(THF)_3$ DMF solution every 6 hours. The solid was washed with oxygen/water-free DMF to get **MIL-100(Sc)-precursor**. The

precursor was solvent exchanged with oxygen/water-free methanol for 3 days before being activated at 150 ^{o}C for 5 hours. After this, the material was exposed to air to get oxidized to **MIL-100(Sc)-Ti.** The color changed from brown to white.

Synthesis of **MOF-74(Zn)**[3]: $Zn(NO_3)_2 \cdot 6H_2O$ (180 mg) and 2,5-dihydroxyterephthalic acid (DOBDC) (70 mg) were dissolved with 15 mL DMF in a 20 mL vial. The vial was sealed and sonicated for 5 minutes until the solid was completely dissolved. To this solution, 1 mL of ethanol followed by 1 mL of deionized water was added dropwise. The vial was sonicated resulting in a clear, yellow solution. This solution was heated in an isothermal oven at 100 °C for 24 h yielding yellow needle crystals **MOF-74(Zn).** Yield (based on ligand): ~80%.

Synthesis of **MOF-74(Zn)-Ti**: As-synthesized 30 mg **MOF-74(Zn)** was washed with dry DMF several times and immersed in dry methanol for 3 days before being activated at 130°C for 9h to remove the terminal solvent molecules on the open metal sites. After activation, **MOF-74(Zn)** was transferred into the glove box when 50 mg $TiCl_3(THF)_3$ and 2mL anhydrous DMF were added. In order to make the exchange complete, the reaction was allowed to continue at 100 ^{o}C for 18 hours. Meantime, the mother liquid was exchanged with fresh $TiCl_3(THF)_3$ DMF solution every 6 hours. The crystals' color changed from light yellow to dark purple. The solid was washed with fresh oxygen/water-free DMF to get **MOF-74(Zn)-precursor**. The precursor was solvent exchanged with oxygen/water-free methanol for 3 days before activated at 60 ^{o}C for 5 hours. After this, the material was exposed to air to get oxidized to dark red **MOF-74(Zn)-Ti.**

Synthesis of **MOF-74(Mg)**[4]: $Mg(NO_3)_2 \cdot 6H_2O$ (150 mg) and DOBDC (60 mg) were dissolved with 15 mL DMF in a 20 mL vial. The vial was sealed and sonicated for 5 minutes until the solid was completely dissolved. To this solution, 1 mL of ethanol and 1 mL of deionized water was added. The vial was sonicated resulting in a clear, light yellow solution. This solution was heated in an isothermal oven at 120 °C for 24 h yielding yellow needle crystals **MOF-74(Mg).** Yield (based on ligand): ~75%.

Synthesis of **MOF-74(Mg)-Ti**: As-synthesized 30 mg **MOF-74(Mg)** was washed with dry DMF several times and immersed in dry methanol for 3 days before being activated at 130°C for 9h to remove the terminal solvent molecules on the open metal sites. The activated **MOF-74(Mg)** was transferred into the glove box when 60 mg $TiCl_3(THF)_3$ in 2mL anhydrous DMF was added. In order to make the exchange complete, the reaction was allowed to continue at 120 ^{o}C for 36 hours. Meantime, the mother liquid was exchanged with fresh $TiCl_3(THF)_3$ DMF solution every 6 hours. The crystals' color changed from yellow to black. The solid was washed with fresh oxygen/water-free DMF to get **MOF-74(Mg)-precursor**. The precursor was solvent exchanged with oxygen/water-free methanol for 3 days before being activated at 60 ^{o}C for 5 hours. After this, the material was exposed to air to get oxidized to orange **MOF-74(Mg)-Ti.**

Section 4: Metal Exchange with PCN-333(Fe) as the Template

To verify the necessity of using template MOFs constructed from redox nonreactive metals, we conducted the same exchange process using PCN-333(Fe) as the template. After $TiCl_3(THF)_3$ was added into PCN-333(Fe) in anhydrous DMF, the crystals totally dissolved resulting in a clear

solution for one minute. Since the redox potential of Fe(III)/Fe(II) is higher than that of Ti(IV)/Ti(III), Fe(III) in the framework was easily reduced to Fe(II), making the framework more labile. Meantime, Ti(IV) species, an extremely strong Lewis acidic species , were generated and they would damage the labile Fe(II) node immediately, further leading to the decomposition of the whole framework.

Section 5: Color Changes during HVMO Process.

The view of the bulk samples and microscopy photos of the templates as well as the corresponding Ti-MOFs were taken. From the figures, we can tell that both the crystal size and shape did not change during the HVMO process, confirming that the metal exchange is a true crystal-to-crystal process. Moreover, in the control experiment, starting from Ti(III) and corresponding ligands, only amorphous products or gel were resulted under the metal exchange condition, suggesting that there is no ligand dissolution and crystal reformation during the HVMO process.

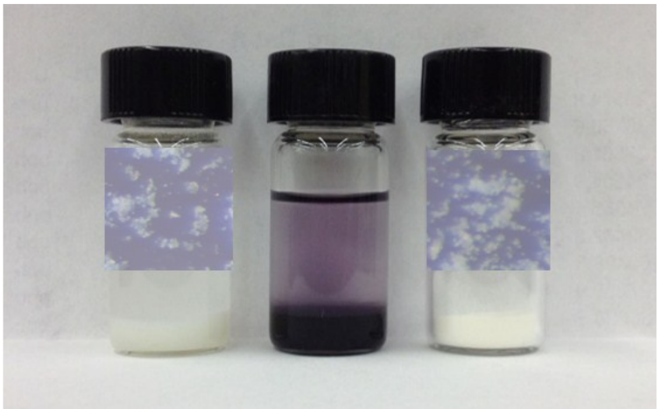

Figure S2. View of the bulk sample for **PCN-333(Sc)**, **PCN-333(Sc)-precursor**, and **PCN-333(Sc)-Ti** (from left to right, the small square inserts are the microscopy photos).

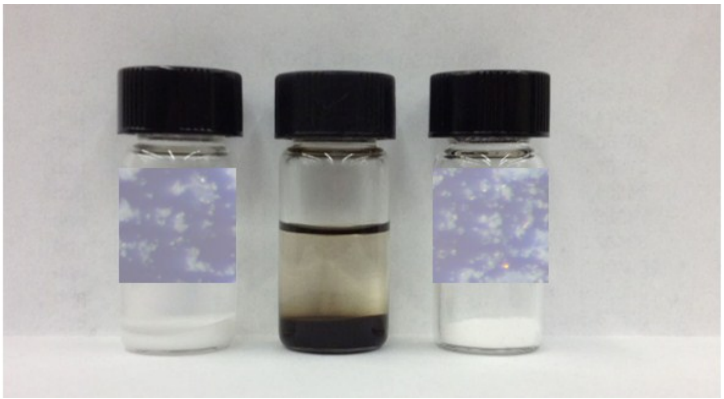

Figure S3. View of the bulk sample for **MIL-100(Sc)**, **MIL-100(Sc)-precursor**, and **MIL-100(Sc)-Ti** (from left to right, the small square inserts are the microscopy photos).

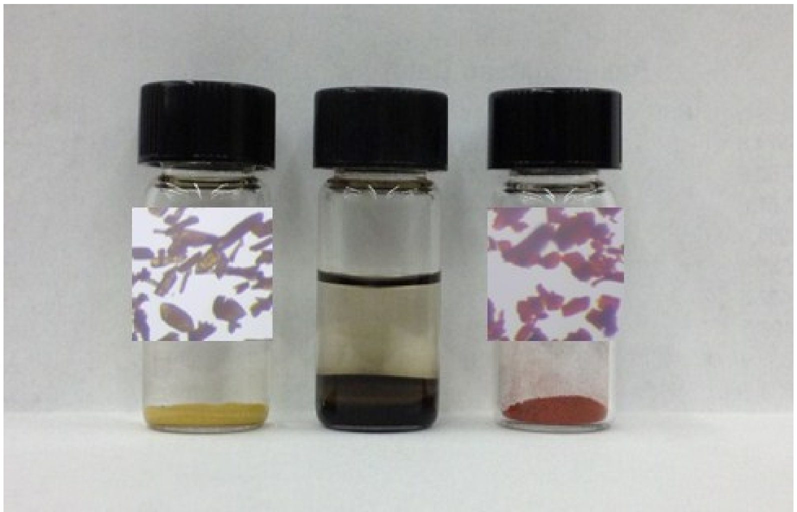

Figure S4. View of the bulk sample for **MOF-74(Zn)**, **MOF-74(Zn)-precursor**, and **MOF-74(Zn)-Ti** (from left to right, the small square inserts are the microscopy photos).

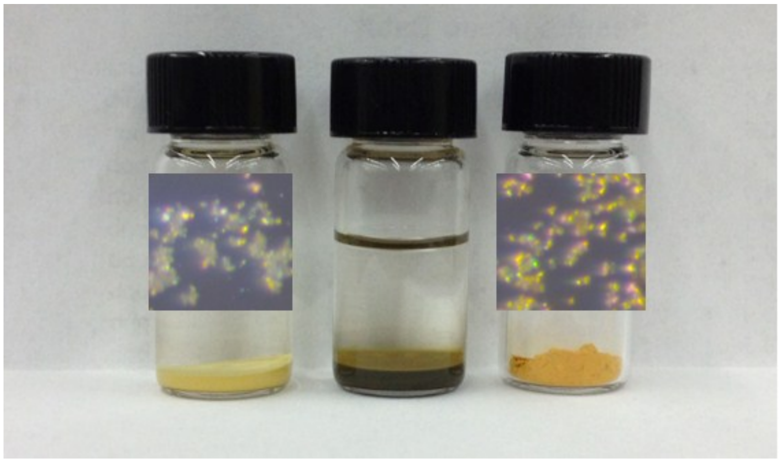

Figure S5. View of the bulk sample for **MOF-74(Mg)**, **MOF-74(Mg)-precursor** and **MOF-74(Mg)-Ti** (from left to right, the small square inserts are the microscopy photos).

Section 6: Powder X-ray Diffraction (PXRD) Patterns for Templates and Ti-MOFs.

Sample preparation: The template MOFs were washed with fresh DMF twice and then washed with acetone three times. After that, these samples were dried in the 65 ^{o}C oven. The corresponding titanium MOFs were obtained after oxidation. The following data was collected on the home device with the λ=1.54178 Å.

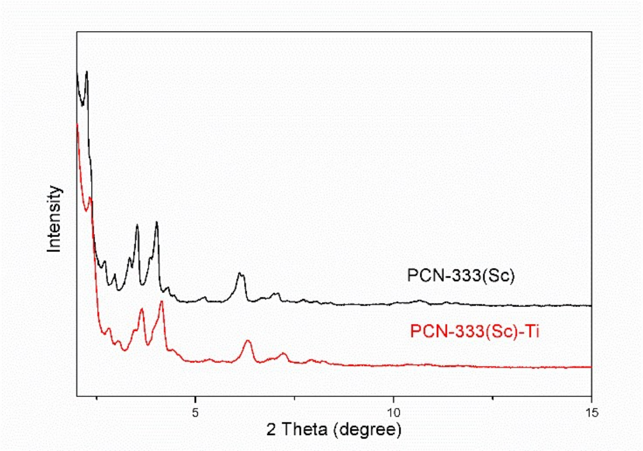

Figure S6. The PXRD of **PCN-333(Sc)** and **PCN-333(Sc)-Ti.**

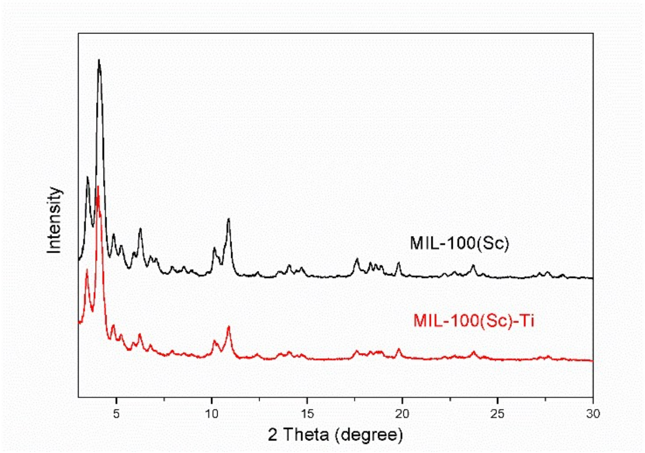

Figure S7. The PXRD of **MIL-100(Sc)** and **MIL-100(Sc)-Ti.**

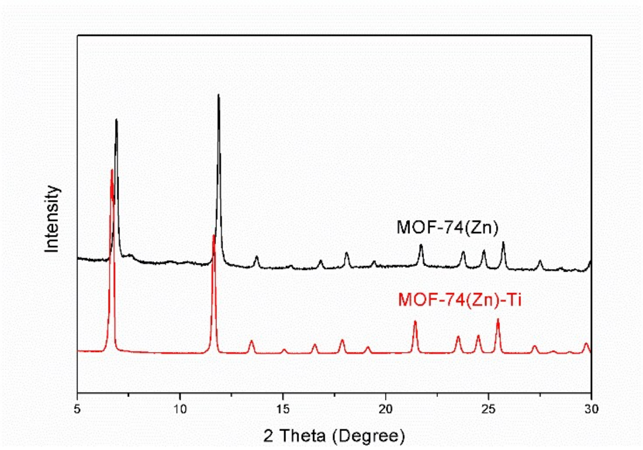

Figure S8.The PXRD of **MOF-74(Zn)** and **MOF-74(Zn)-Ti.**

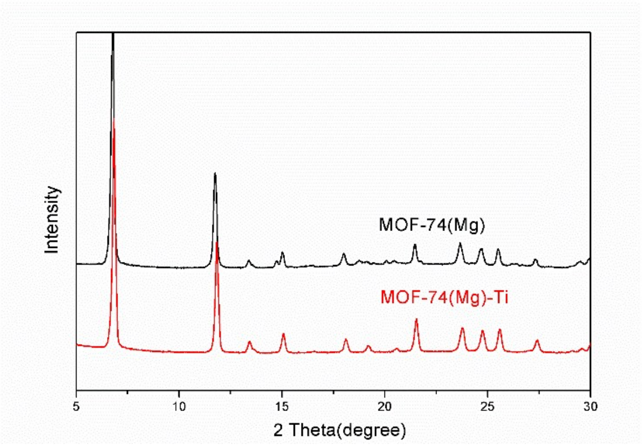

Figure S9. The PXRD of **MOF-74(Mg)** and **MOF-74(Mg)-Ti.**

Section 7: Powder Refinements for MOF-74(Zn)-Ti and MOF-74(Mg)-Ti.

The high resolution synchrotron PXRD data files of MOF-74(Zn), MOF-74(Zn)-Ti, MOF-74(Mg) and MOF-74(Mg)-Ti were collected at beamline 17-BM at the Advanced Photon Source, Argonne National Laboratory (Figure S10, S11) using the wavelength of 0.72768 Å. The structure model of **MOF-74(Zn)-Ti** was built based on the reported MOF-74(Zn) structure possessing R $\bar{3}$ space group. The Rietveld refinement was performed using *TOPAS Academic version 4.2*. The background was modelled with a 7th order Chebychev polynomial, ranging from 2° to 21.5°. The whole pattern was corrected with respect to zero shift and specimen displacement. The unit cell parameters were refined and the peak profile was fitted using a PV-TCHZ peak type. As a result, R-value was converged to R_p=3.74, R_{wp}=2.60, R_{exp}=2.22, and GOF=1.69. The structure model of **MOF-74(Mg)-Ti** was refined as the similar procedure described above as well. All the crystallographic results were summarized in Table S1 and the unit cell parameters were summarized in Table S2. From the tables, we can tell that MOF-

74(Zn), MOF-74(Zn)-Ti, MOF-74(Mg) and MOF-74(Mg)-Ti have very similar unit cells and they all share the MOF-74 structure.

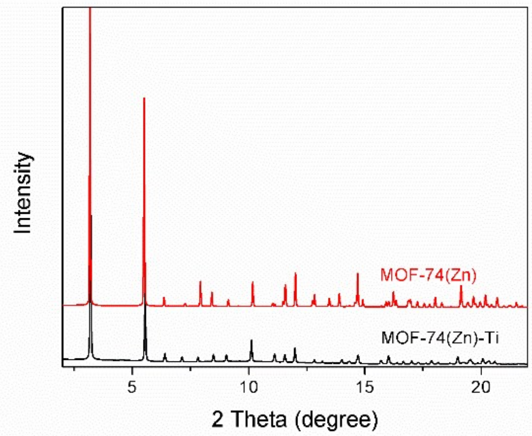

Figure S10.The synchrotron PXRD of **MOF-74(Zn)** and **MOF-74(Zn)-Ti.**

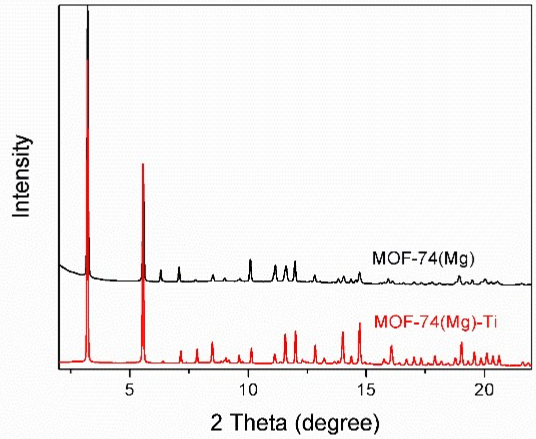

Figure S11.The synchrotron PXRD of **MOF-74(Mg)** and **MOF-74(Mg)-Ti.**

Table S1. Crystallographic data and results for the Rietveld refinement of MOF-74(Mg)-Ti and MOF-74(Zn)-Ti.

	**MOF-74(Mg)-Ti**	**MOF-74(Zn)-Ti**
Space group	$R\bar{3}$	$R\bar{3}$
a (Å)	25.9260000	26.2107146
c (Å)	6.9150000	6.6547168
Cell Volume (Å^3)	4025.25918	3959.29562
Temperature (K)	295	295
Wavelength (Å)	0.7276	0.7276
2θ range (degree)	2.0~15.0	2.0~21.5
**R_p**	1.52	2.60
**R_{wp}**	1.96	3.74
**R_{exp}**	1.85	2.22
**GoF**	1.06	1.69

Table S2. The comparison of unit cells for MOF-74.

	Spacegroup	a (Å)	c (Å)
MOF-74(Zn)	$R\bar{3}$	25.9322	6.8365
MOF-74(Zn)-Ti	$R\bar{3}$	26.2107	6.6547
MOF-74(Mg)	$R\bar{3}$	26.0261	6.7587
MOF-74(Mg)-Ti	$R\bar{3}$	25.9260	6.9150

Section 8: Porosity Measurements for the Template MOFs and Ti-MOFs.

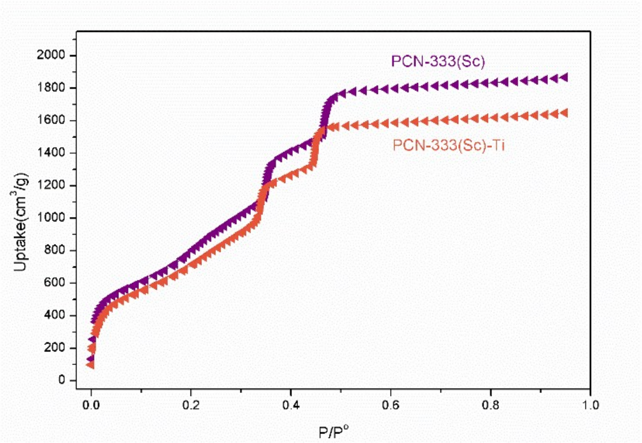

Figure S12. The N_2 adsorption isotherms of **PCN-333(Sc)**, and **PCN-333(Sc)-Ti** at 77 K, 1 atm**.**

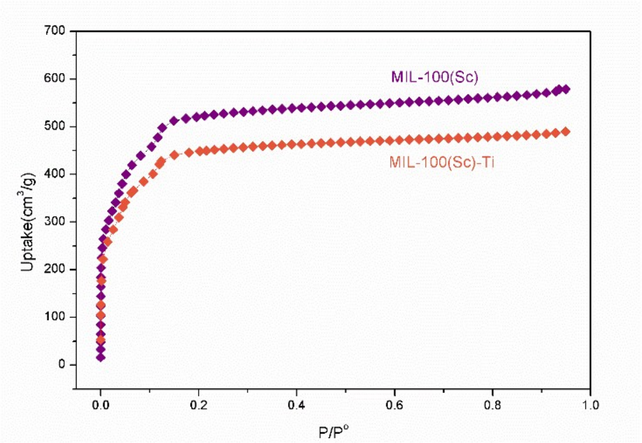

Figure S13. The N_2 adsorption isotherms of **MIL-100(Sc)**, and **MIL-100(Sc)-Ti** at 77 K, 1 atm**.**

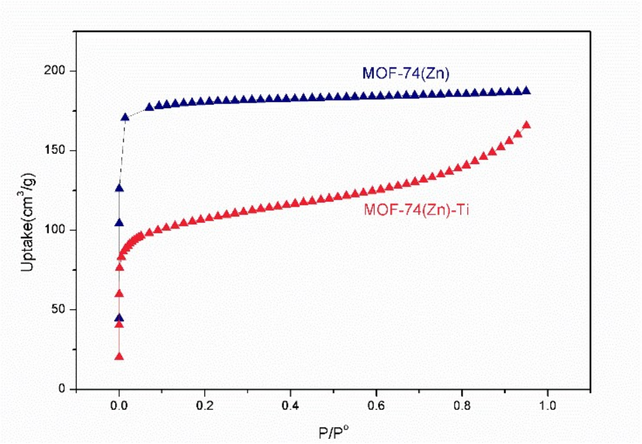

Figure S14. The N_2 adsorption isotherms of **MOF-74(Zn)**, and **MOF-74(Zn)-Ti** at 77 K, 1 atm**.**

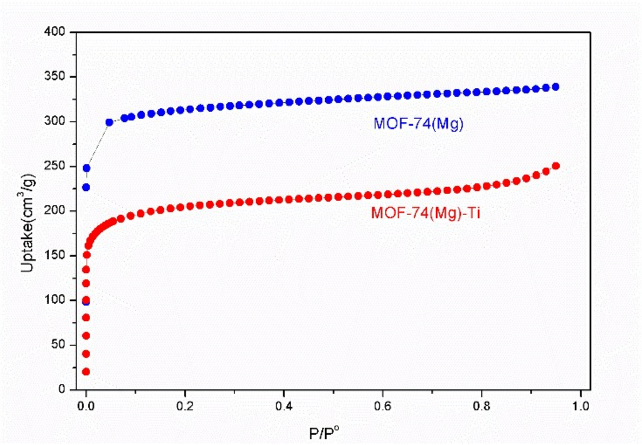

Figure S15. The N_2 adsorption isotherms of **MOF-74(Mg)** and **MOF-74(Mg)-Ti** at 77 K, 1 atm**.**

Section 9. Energy Dispersive X-ray Spectroscopy (EDS) and Inductively Coupled Plasma Emission-Mass Spectrometry (ICP-MS) Analysis.

Element	Weight%	Atomic%
C K	48.69	63.19
O K	30.11	29.33
Cl K	4.59	2.02
Sc K	2.23	0.77
Ti K	14.38	4.68
Totals	100.00	

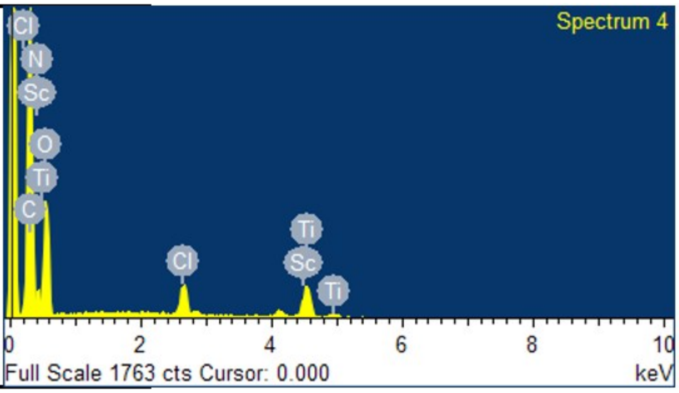

Figure S16. EDS for **PCN-333(Sc)-Ti.**

Element	Weight%	Atomic%
C K	30.85	44.37
O K	41.45	44.76
Cl K	4.98	2.43
Sc K	10.54	4.05
Ti K	12.19	4.39
Totals	100.00	

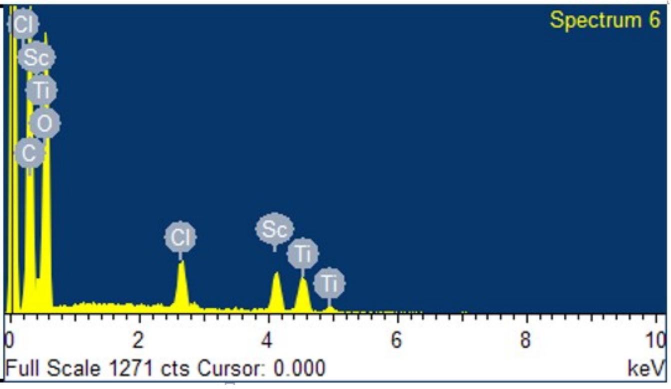

Figure S17. EDS for **MIL-100(Sc)-Ti**

Element	Weight%	Atomic%
C K	39.00	51.42
O K	43.10	42.66
Ti K	17.89	5.92
Totals	100.00	

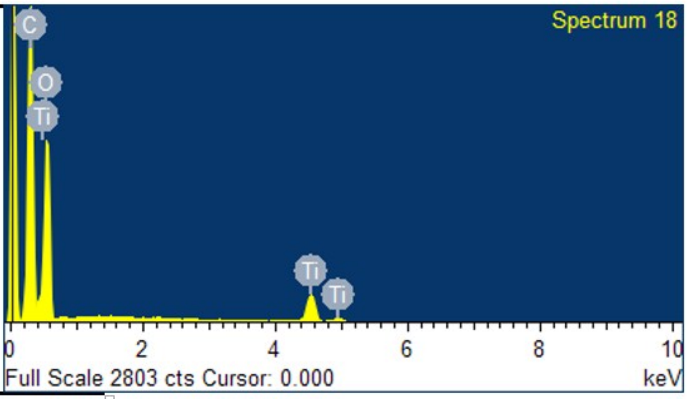

Figure S18. EDS for **MOF-74(Zn)-Ti**

Element	Weight%	Atomic%
C K	56.81	67.14
O K	31.10	27.60
Mg K	5.85	3.42
Ti K	6.24	1.85
Totals	100.00	

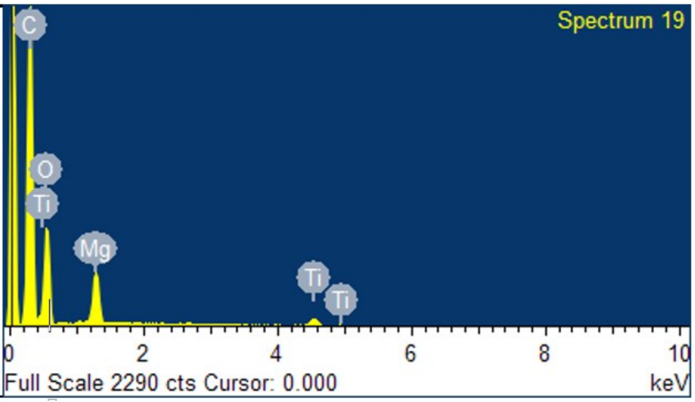

Figure S19. EDS for **MOF-74(Mg)-Ti**

To demonstrate that Ti was successfully exchanged into the framework, EDS measurement using copper substrate was performed (Figure S20). The structure formula of MOF-74(Mg) is $(C_8H_6Mg_2O_8)n,8(H_2O)n$, showing the atomic ratio of C/Mg = 4. Hence, the theoretical atomic ratio of C/(Mg+Ti) after metal exchange should be 4. From the EDS result, we can tell that the experimental atomic rat io of C/(Mg+Ti) = 4.2, which is very consistent to theoretical calculation, suggesting no depositing of Ti species in the framework.

Element	Weight%	Atomic%
C K	26.26	36.33
O K	51.16	55.12
Mg K	8.09	5.53
Ti K	14.49	3.03
Totals	100.00	

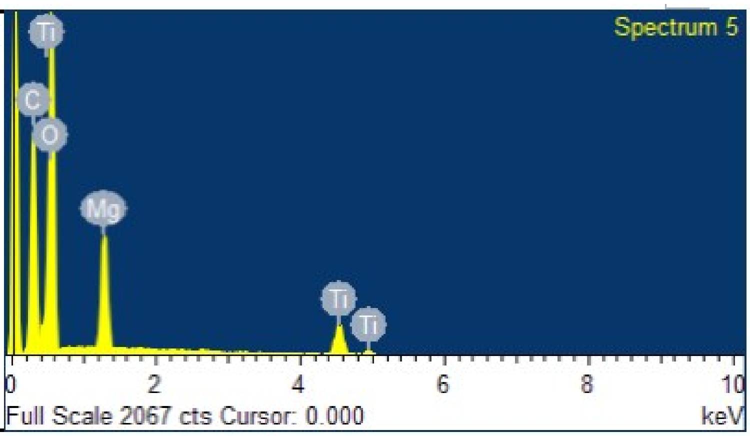

Figure S20. EDS for MOF-74(Mg)-Ti using copper substrate showing that the ratio of C/(Mg+Ti)~4, proving that Ti was surely exchange into the framework.

In the sample preparation for the ICP test, a 3 mg sample was added into 1 mL of concentrated HNO_3, and heated to 70 ^oC for 4 hours while sonicating. By then, the MOFs had been completely decomposed and metal ions had been dissolved in the aqueous solution. To prove this, the solid residue after the sample preparation was added into DMF and there was no insoluble residue left, indicating the MOFs had been completely dissolved. Moreover, the ICP results in the main text is the average of three independent measures. Therefore it is believed that the ICP results are accurate.

Table S3. Inductively Coupled Plasma Emission-Mass Spectrometry

	Sample	Sc/Zn/Mg (ppb)	Ti (ppb)	Ti percent (atomic)
**PCN-333(Sc)-Ti**	1st Batch	254	2021	88.2%
	2nd Batch	328	2528	87.9%
	3rd Batch	228	1766	87.9%
**MIL-100(Sc)-Ti**	1st Batch	706	864	53.5%
	2nd Batch	893	858	47.4%
	3rd Batch	925	822	45.5%
**MOF-74(Zn)-Ti**	1st Batch	113	1096	93.0%
	2nd Batch	89	1458	95.7%

	3rd Batch	73	1134	95.5%
**MOF-74(Mg)-Ti**	1st Batch	279	353	38.8%
	2nd Batch	189	226	37.5%
	3rd Batch	138	164	37.3%

Section 10. Water Stability Test for Ti-MOFs.

The pristine Ti-MOFs were soaked in pure water for certain time. After being washed with acetone 3 times, these samples were dried in the 65 °C oven. Then the following PXRD data was collected on the home device with λ=1.54178 Å. From the PXRD results, we can tell that MIL-100(Sc)-Ti, MOF-74(Zn)-Ti, and MOF-74(Mg)-Ti can stay in pure water for 18h without losing their crystallinity. PCN-333(Sc)-Ti only maintains its crystallinity in pure water for 3h probably due to the much larger ligand and porosity. Such extended linker effects on the framework stability has also been reported in many other MOF systems, such as UiO-66, 67, 68[5].

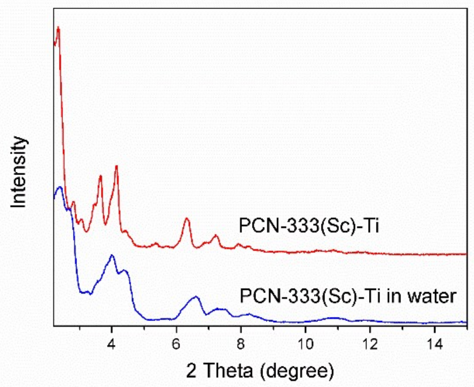

Figure S21. The PXRD patterns of pristine **PCN-333(Sc)-Ti** and **PCN-333(Sc)-Ti** treated with water for 3h.

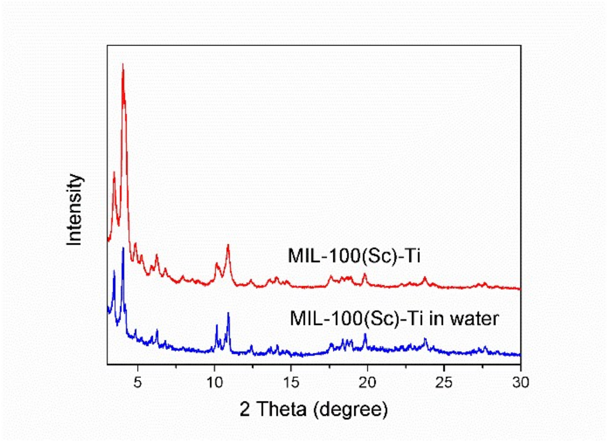

Figure S22. The PXRD patterns of pristine **MIL-100(Sc)-Ti** and **MIL-100(Sc)-Ti** treated with water for 18h.

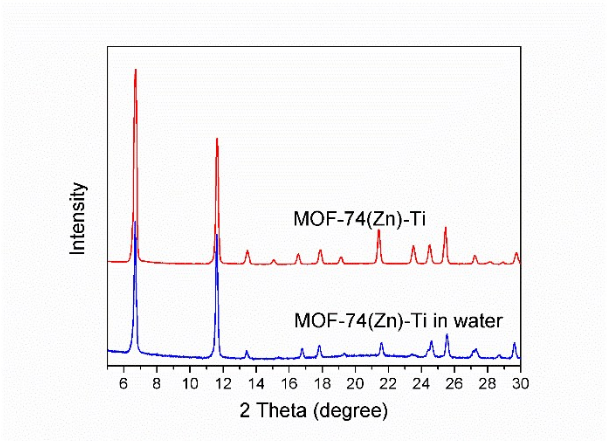

Figure S23. The PXRD patterns of pristine **MOF-74(Zn)-Ti** and **MOF-74(Zn)-Ti** treated with water for 18h.

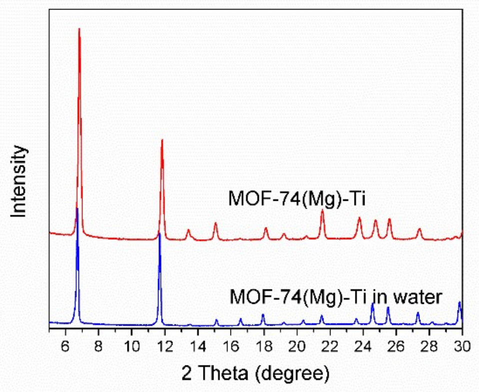

Figure S24. The PXRD patterns of pristine **MOF-74(Mg)-Ti** and **MOF-74(Mg)-Ti** treated with water for 18h.

Section 11. Optimization of Exchange Conditions for PCN-333(Sc)-Ti.

PCN-333(Sc)-Ti was taken as an example to illustrate how elongation of reaction time would affect the exchange process. Please check the detail exchange procedure in Section 3. The exchange reaction was stopped at 6h, 12h, 24h, 30h, respectively. After the regular work up procedure, the titanium atomic ratio in the products was characterized using ICP-MS measurement. According to our results, when the exchange time increase, the exchange ratio increases. However, the increment of the exchange ratio gradually slowed down. On the other hand, the destruction of the framework continues along with the exchange process. To avoid significant crystallinity destruction, we stopped the exchange reaction at a balance point, where both high exchange ratio and framework crystallnity can be guaranteed.

Table S4. Element Analysis of PCN-333(Sc)-Ti by elongating the reaction time.

	120°C_6h	120°C_12h	120°C_24h	120°C_30h
EDS	50.0%	66.7%	85.9%	88.7%
ICP-MS	53.5%	67.2%	88.0%	88.1%

In our experiment, we already used the highest temperature the exchange condition allowed. Since our template MOFs are relative stable, higher exchange temperature is preferred to overcome the high activation energy. However, the temperature cannot be too high for the following reasons: Firstly, Ti(III) is more easily oxidized at high temperature. Secondly, our exchange temperature is the same with or just slightly lower than the one our template MOFs were synthesized in. When the temperature goes higher, our template MOFs can be more easily attacked by the free cations in the solution, which could cause dramatic damage to the framework crystallinity. So we chose 120 ^{o}C as exchange temperature and 24h as exchange time for PCN-333(Sc)-Ti.

Section 12. X-ray Photoelectron Spectroscopy (XPS) Analysis of Ti-MOFs.

In order to confirm the oxidation state of products, we performed XPS measurement for all the Ti-MOFs. Figure S25 represents the binding energy of Ti 2p region for PCN-333(Sc)-Ti. The Ti2$p_{3/2}$ signal at 458.7 eV and Ti2$p_{1/2}$ signal at 464.2 eV well indicate the Ti^{4+} species in the sample. In the same way, the Ti^{4+} species can be confirmed for MIL-100(Sc)-Ti (Figure S26), MOF-74(Zn)-Ti (Figure S27), and MOF-74(Mg)-Ti (Figure S28), which conclude the successful oxidation of the metal nodes.

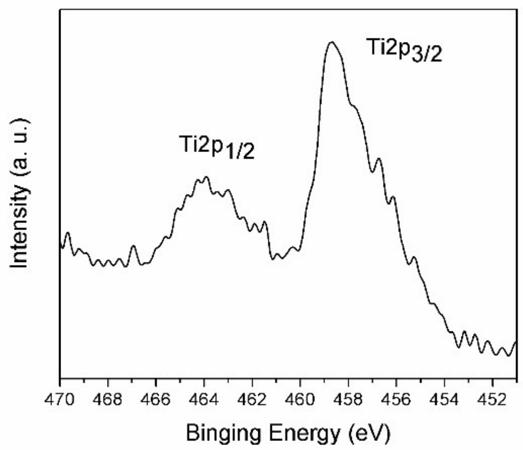

Figure S25. XPS spectra of PCN-333(Sc)-Ti.

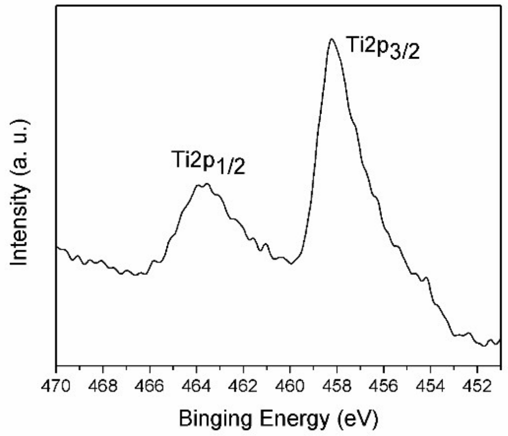

Figure S26. XPS spectra of MIL-100(Sc)-Ti.

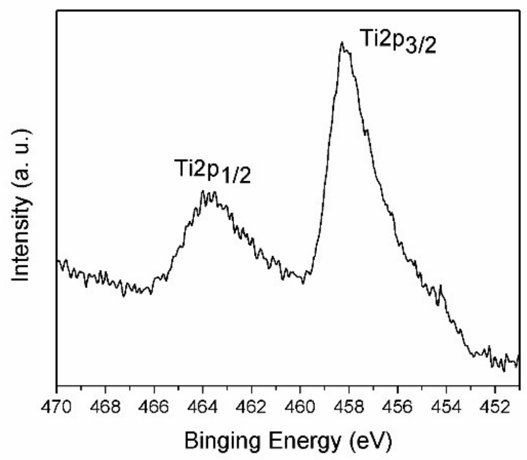

Figure S27. XPS spectra of MOF-74(Zn)-Ti.

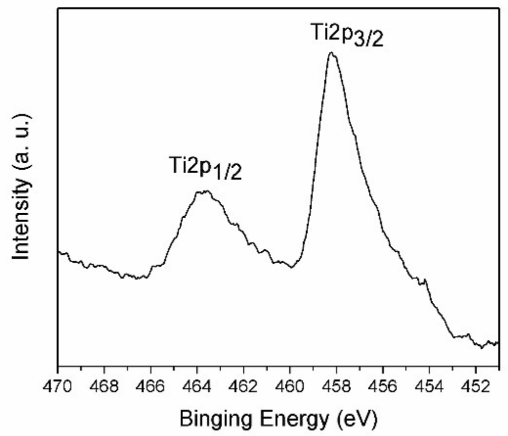

Figure S28. XPS spectra of MOF-74(Mg)-Ti.

Section 13. Photoabsorption Performance of Ti-MOFs.

The calculated optical bandgap of **MOF-74(Zn)-Ti** from the absorption onset is 2.02 eV. It should be pointed out that **MOF-74(Zn)-Ti** is composed of discrete titanium-oxo clusters which are

separated by organic ligands. The interaction between these clusters is not strong enough to form a continuous band. This makes Ti-MOFs different from classical semiconductors.

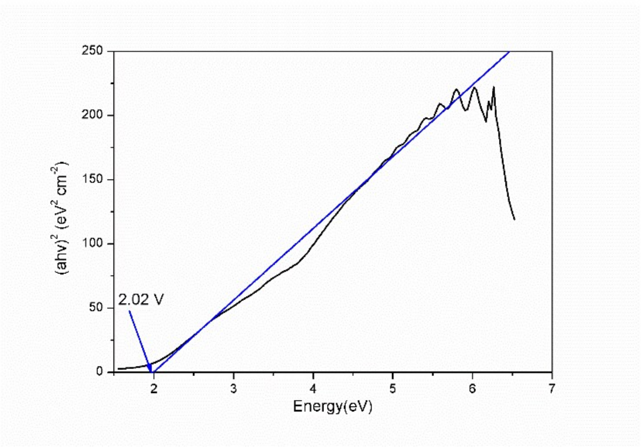

Figure S29. Band gap calculation from UV-Vis spectra of **MOF-74(Zn)-Ti**.

Section 14. The Photoelectrochemical Properties of MOF-74(Zn)-Ti.

Electrode preparation. To prepare the photoelectrodes, 10 mg of **MOF-74(Zn)-Ti** was first ground using a marble mortar and pestle, and then added into 2 ml of acetone. The as-prepared solution was sonicated for one hour. Meanwhile, the fluorine-doped tin oxide (FTO) substrates were cleaned thoroughly by sonication in 5% detergent for 30 min first and then rinsed with deionized water for several times, which were followed by sonication in deionized water for 15 min three times. After that, 0.5 mL of the 5 mg/ml **MOF-74(Zn)-Ti** solution was dropped onto the surface of FTO substrate, which was masked by a scotch tape with an exposed area of 2.0 × 0.5 cm^2, and then dried in air at 70˚C in the oven.

Photoelectrochemical measurements. The photoelectrochemical tests were performed using an electrochemical workstation (CHI 830b). The photocurrent test was carried out using a two-electrode setup, in which the working electrode (**MOF-74(Zn)-Ti** /FTO electrode) and the counter electrode (Pt plate electrode) were short-circuited. The 0.5 M Na_2SO_4 solution was used as the electrolyte. A 30 W fluorescent light bulb (> 450 nm) was used as the visible light source. The Mott-Schottky curves were measured in dark using a three-electrode cell at frequencies of 10 Hz. The Pt plate was used as a counter electrode and the Ag/AgCl electrode (3 M KCl) was used as reference electrode. Prior to each measurement, the electrolyte was deaerated by purging it with nitrogen continuously for 30 minutes.

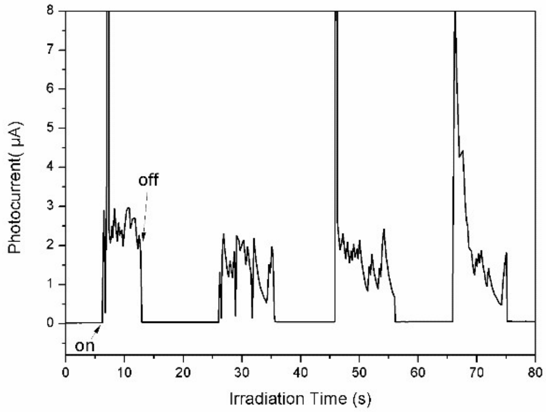

Figure S30. Transient photocurrent response of **MOF-74(Zn)-Ti** in 0.5 M Na_2SO_4 aqueous solution without bias versus Ag/AgCl under the irradiation of visible light.

Figure S30 shows the transient photocurrent responsed of **MOF-74(Zn)-Ti** under intermittent visible light illumination. The result indicates that **MOF-74(Zn)-Ti** is able to be photoexcited to generate electron-hole pairs and these photogenerated carriers can be efficiently separated

upon contact with a high work function conductor under visible light irradiation, which further proves the feasibility of **MOF-74(Zn)-Ti** as a visible light driven photocatalyst.

Figure S31 displays the capacitance measurements in the Mott-Schottky-type plot for **MOF-74(Zn)-Ti.** The positive slope of the obtained C^{-2}-E plot is consistent with that of typical n-type semiconductor. Another important parameter derived from this measurement is the flat-band potential. It can be obtained that the flat-band potential of **MOF-74(Zn)-Ti** is around -0.57 V vs. Ag/AgCl. According to the calculated band gap 2.02 V, the conduction band potential is equal to 1.45 V vs. Ag/AgCl.

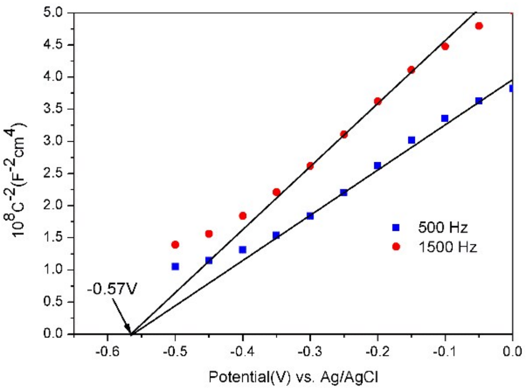

Figure S31. Mott-Schottky plot of **MOF-74(Zn)-Ti** in 0.5 M Na_2SO_4 aqueous solution (pH=7.0).

Section 15. Photodegradation of Methylene Blue with Ti-MOFs.

Photocatalytic experiments: The evaluation of photocatalytic activities of the samples for the photocatalytic degradation of organic dyes was performed at ambient temperature (25 ℃). The procedure was as follows: 15mg of sample was dispersed into 15 mL of methylene blue (MB)

aqueous solution (500 µmol L^{-1}). The mixture was stirred continuously with a magnetic stirring bar for two hours in the dark to reach the adsorption equilibrium. The photocatalytic dye degradation was carried out by irradiating the suspension with a 300 W Xenon lamp. At different time intervals, analytical samples were withdrawn and analyzed by UV-Vis spectroscopy. The degradation efficiency was determined by dividing C/C_0 with time, where C is the remained MB concentration and C_0 is the starting MB concentration.

From the following figures, we can tell that all the Ti-MOFs showed much better catalytic performance than the corresponding template MOFs, indicating the Ti content in our Ti-MOFs indeed play a vital role in the photocatalysis process.

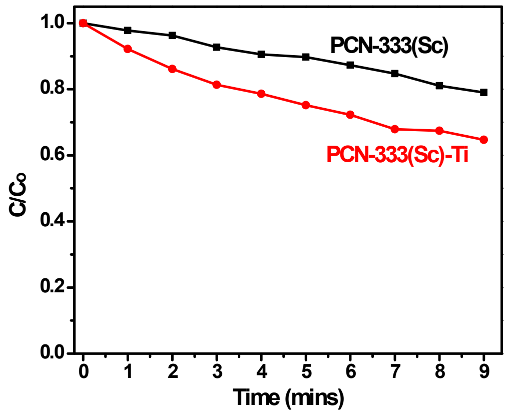

Figure S32. Photodegradation of MB using PCN-333(Sc) and PCN-333(Sc)-Ti.

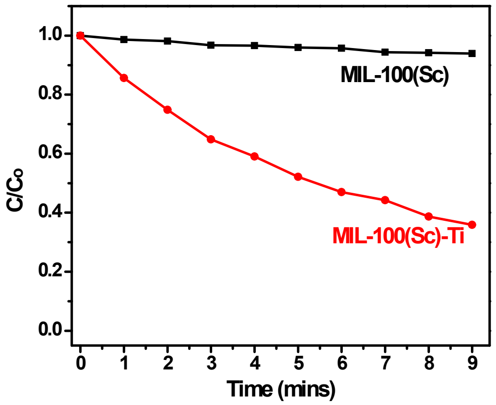

Figure S33. Photodegradation of MB using MIL-100(Sc) and MIL-100(Sc)-Ti.

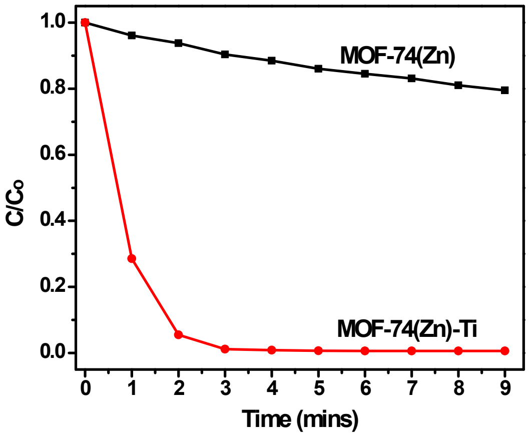

Figure S34. Photodegradation of MB using MOF-74(Zn) and MOF-74(Zn)-Ti.

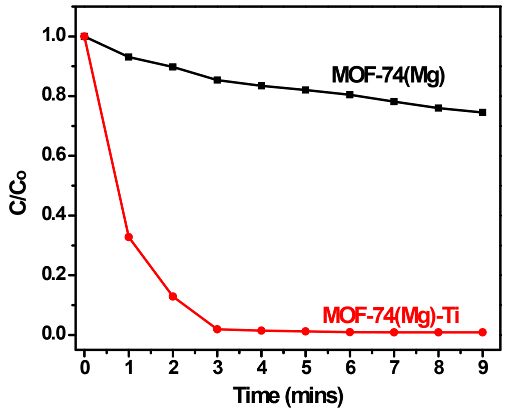

Figure S35. Photodegradation of MB using MOF-74(Mg) and MOF-74(Mg)-Ti.

Section 16. PXRD Patterns of Ti-MOFs after MB Photodegradation.

After the MB photodegradation experiment, the samples were washed with acetone 3 times, and then dried in the 65 ^{o}C oven. The following PXRD data was collected on the home device with λ=1.54178 Å. All Ti-MOFs maintain their crystallinity well during the MB photodegradation process.

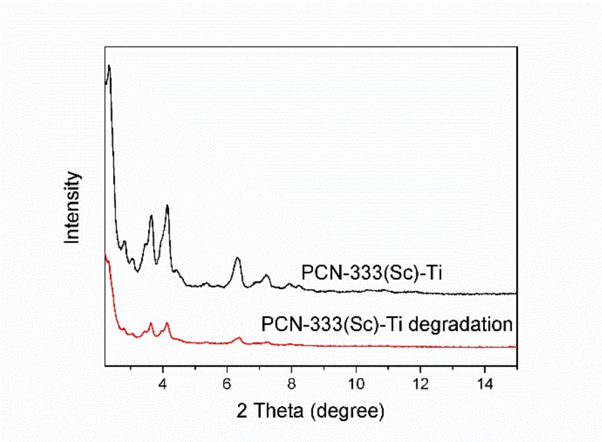

Figure S36. The PXRD patterns of pristine **PCN-333(Sc)-Ti** and **PCN-333(Sc)-Ti** after MB degradation.

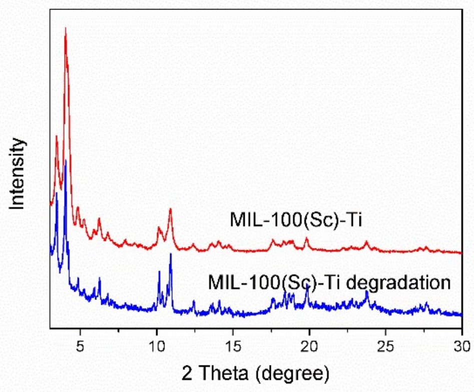

Figure S37. The PXRD patterns of pristine **MIL-100(Sc)-Ti** and **MIL-100(Sc)-Ti** after MB degradation.

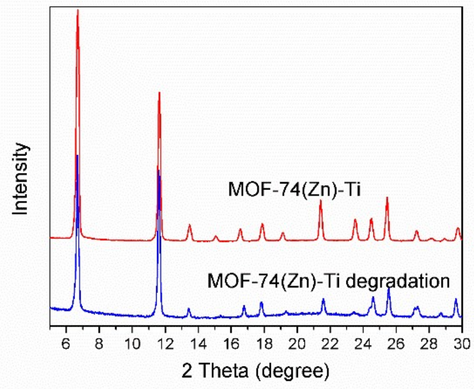

Figure S38. The PXRD patterns of pristine **MOF-74(Zn)-Ti** and **MOF-74(Zn)-Ti** after MB degradation.

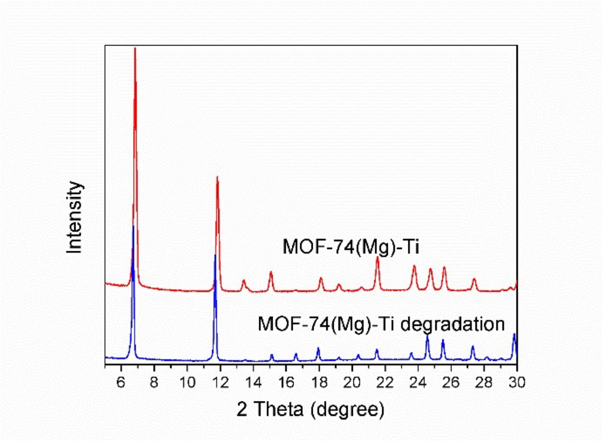

Figure S39. The PXRD patterns of pristine **MOF-74(Mg)-Ti** and **MOF-74(Mg)-Ti** after MB degradation.

Section 17. Thermogravimetric Analysis.

About 10 mg of sample was heated on a TGA-50 (Shimadzu) thermogravimetric analyzer from room temperature to 700 °C at a rate of 3 °C·min^{-1} under N_2 flow of 25 mL·min^{-1}.

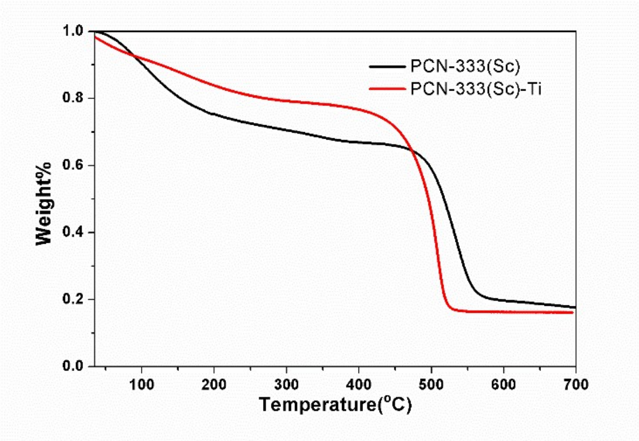

Figure S40. TGA curves for **PCN-333(Sc)**, and **PCN-333(Sc)-Ti**.

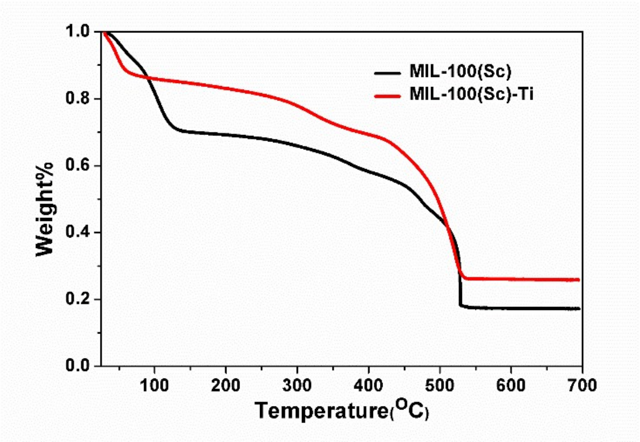

Figure S41. TGA curves for **MIL-100(Sc)**, and **MIL-100(Sc)-Ti**.

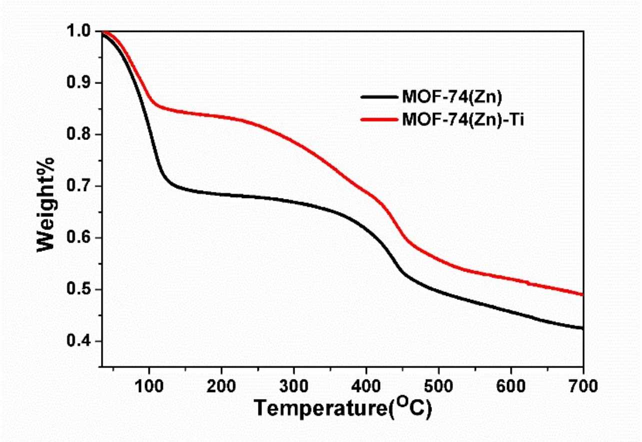

Figure S42. TGA curves for **MOF-74(Zn)** and **MOF-74(Zn)-Ti**.

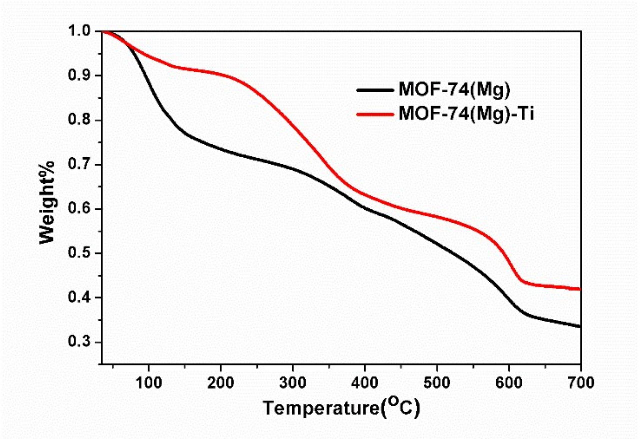

Figure S43. TGA curves for **MOF-74(Mg)** and **MOF-74(Mg)-Ti**.

Reference:

[1] D. Feng, K. Wang, Z. Wei, Y.-P. Chen, C. M. Simon, R. K. Arvapally, R. L. Martin, M. Bosch, T.-F. Liu, S. Fordham, D. Yuan, M. A. Omary, M. Haranczyk, B. Smit, H.-C. Zhou, *Nat. Commun.* **2014**, *5*.

[2] L. Mitchell, B. Gonzalez-Santiago, J. P. S. Mowat, M. E. Gunn, P. Williamson, N. Acerbi, M. L. Clarke, P. A. Wright, *Catal. Sci. Technol.* **2013**, *3*, 606-617.

[3] S. J. Geier, J. A. Mason, E. D. Bloch, W. L. Queen, M. R. Hudson, C. M. Brown, J. R. Long, *Chem. Sci.* **2013**, *4*, 2054-2061.

[4] H. Deng, S. Grunder, K. E. Cordova, C. Valente, H. Furukawa, M. Hmadeh, F. Gándara, A. C. Whalley, Z. Liu, S. Asahina, H. Kazumori, M. O'Keeffe, O. Terasaki, J. F. Stoddart, O. M. Yaghi, *Science* **2012**, *336*, 1018-1023.

[5] Cavka, J. H.; Jakosen, S.; Olsbye, U.; Guillou, N.; Lamberti, C.; Bordiga, S.; Lillerud, K. P., *J. Am. Chem. Soc.* **2008**, *130*(42), 13850-13851.
